# Fish Health Altered by Contaminants and Low Water Temperatures Compounded by Prolonged Regional Drought in the Lower Colorado River Basin, USA

**DOI:** 10.3390/toxics12100708

**Published:** 2024-09-28

**Authors:** Steven L. Goodbred, Reynaldo Patiño, David A. Alvarez, Darren Johnson, Deena Hannoun, Kathy R. Echols, Jill A. Jenkins

**Affiliations:** 1 U.S. Geological Survey, California Water Science Center, Sacramento, CA 95819, USA; sgoodbred@usgs.gov; 2 U.S. Geological Survey, Texas Cooperative Fish & Wildlife Research Unit and Departments of Natural Resources Management and of Biological Sciences, Lubbock, TX 79409, USA; reynaldo.patino@ttu.edu; 3 U.S. Geological Survey, Columbia Environmental Research Center, Columbia, MO 65201, USA; dalvarez@usgs.gov (D.A.A.); kechols@usgs.gov (K.R.E.); 4 U.S. Geological Survey, Wetland and Aquatic Research Center, Lafayette, LA 70506, USA; johnsond@contractor.usgs.gov; 5 Southern Nevada Water Authority, Las Vegas, NV 89106, USA; deena.hannoun@lvwd.com

**Keywords:** environmental organic contaminants, sperm quality, gonad histology, estrogenicity, climate change

## Abstract

The goal of this study was to assess health of male Common Carp (carp, *Cyprinus carpio*) at four sites with a wide range in environmental organic contaminant (EOC) concentrations and water temperatures in Lake Mead National Recreation Area NV/AZ, US, and the potential influence of regional drought. Histological and reproductive biomarkers were measured in 17–30 carp at four sites and 130 EOCs in water per site were analyzed using passive samplers in 2010. Wide ranges among sites were noted in total EOC concentrations (>10Xs) and water temperature/degree days (10Xs). In 2007/08, total polychlorinated biphenyls (tPCBs) in fish whole bodies from Willow Beach (WB) in the free-flowing Colorado River below Hoover Dam were clearly higher than at the other sites. This was most likely due to longer exposures in colder water (12–14 °C) and fish there having the longest lifespan (up to 54 years) for carp reported in the Colorado River Basin. Calculated estrogenicity in water exceeded long-term, environmentally safe criteria of 0.1–0.4 ng/L by one to three orders of magnitude at all sites except the reference site. Low ecological screening values for four contaminants of emerging concern (CEC) in water were exceeded for one CEC in the reference site, two in WB and Las Vegas Bay and three in the most contaminated site LVW. Fish health biomarkers in WB carp had 25% lower liver glycogen, 10Xs higher testicular pigmented cell aggregates and higher sperm abnormalities than the reference site. Sperm from LVW fish also had significantly higher fragmentation of DNA, lower motility and testis had lower percent of spermatozoa, all of which can impair reproduction. Projections from a 3D water quality model performed for WB showed that EOC concentrations due to prolonged regional drought and reduced water levels could increase as high as 135%. Water temperatures by late 21st century are predicted to rise between 0.7 and 2.1 °C that could increase eutrophication, algal blooms, spread disease and decrease dissolved oxygen over 5%.

## 1. Introduction

**Figure 1:**
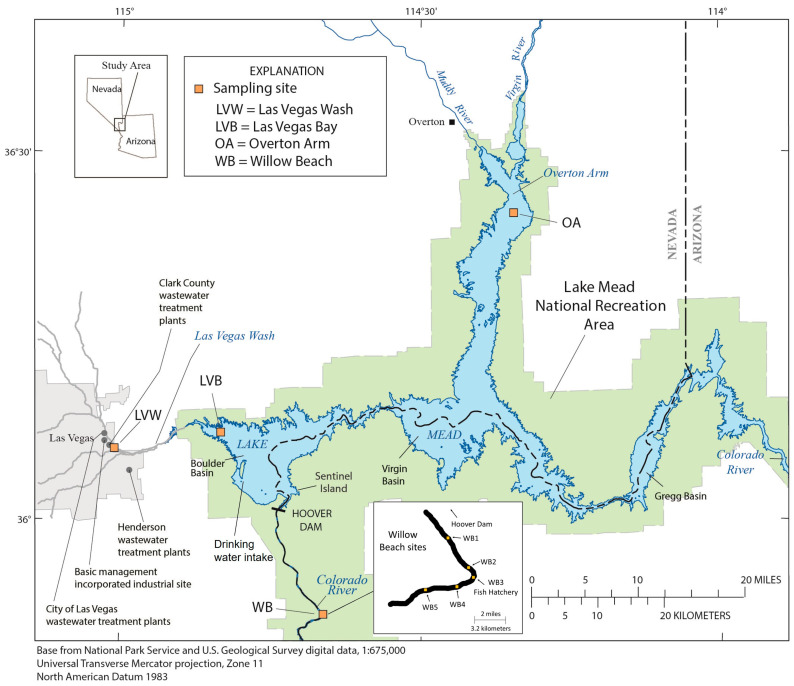
Location of the four sampling sites in and near the Lake Mead National Recreation Area (LMNRA), NV and AZ, US where Common Carp (*Cypinus carpio*) were collected, and semipermeable membrane device samplers (SPMD) deployed. The Willow Beach inset shows the five sites in the Colorado River below Hoover Dam where sediment and periphyton were collected and SPMDs were deployed to assess potential polychlorinated biphenyl sources. Lake Mead formed by the Hoover Dam is within the recreation area (green area). Note the drinking water withdrawal location for the City of Las Vegas in Boulder Basin below Las Vegas Wash where there are three sewage treatment plants and an industrial site in Henderson with surface and underground contamination [4]. The reference site is Overton Arm, at the northern part of LMNRA.

Lake Mead National Recreation Area (LMNRA) in the Colorado River Basin (CRB) was the first national recreation area created in the United States (U.S.) in 1936 and has the largest area (6053 km^2^) and largest reservoir, Lake Mead, by volume (max 34.84 km^3^) (Figure 1) [1]. It is a popular destination for fishing, boating, hiking, and camping, and is close to the City of Las Vegas with a metropolitan population of nearly three million people [2]. Diverse aquatic ecosystems found in riverine sections of the free-flowing Colorado River above and below Lake Mead provide a variety of habitats and shallow waters with aquatic vegetation and deep-water benthos. LMNRA also provides water for 25 million people for urban, industrial, agriculture uses, and hydroelectric power for 1.3 million people in the Southwest U.S. [3]. Below Hoover Dam, the hypolimnetic water released from Lake Mead establishes a ≈21 km stretch of free-flowing river before entering Lake Mohave where unusually cool water temperatures exist within a narrow range of 12–15 °C throughout the year [4].

Lakes Mead and Mohave provide a rich, diverse ecosystem influenced by non-native species, including golden algae (*Prymnesium parvum*) and three mollusks, including New Zealand mud snail (*Potamopyrgus antipodarium*), Asian basket clam (*Corbicula fluminea*), and quagga mussel (*Dreissena rostriformis*) [5]. Of these invasives, quagga mussels are the most destructive because they colonize in vast numbers on hard surfaces (i.e., biofouling drinking water intakes), and they have established substantial populations since their accidental introduction to Lake Mead in 2007 [6]. With large numbers of quagga mussels in Lake Mead (estimated at 1.5 × 10^12^ in 2014), with each mussel able to filter ≈1 L water per day, they remove approximately10.5% of environmental organic contaminants (EOCs) from the water column to the benthic ecosystem [7].

Although most of Lake Mead has generally good water quality for nutrients and dissolved oxygen [8], there are several threats to the federally listed Razorback Sucker (*Xyrauchen texanus*) due to non-native fish previously introduced to establish a sport fishery, reduced habitat from lower lake levels, and increasing regional population growth [9]. Additional stressors, including EOCs in LMNRA, have been previously discussed [4], where the areas of most historical concern are Las Vegas Wash (LVW), Las Vegas Bay (LVB) and Willow Beach (WB) (Figure 1). The LVW is the main drainage for natural and urban runoff from the Las Vegas Valley, that consists of ≈85% wastewater effluent [10]. The stream channel is adjacent to the Black Mountain Industrial complex in Henderson, NV that was developed after World War II for various industrial chemical companies. Early disposal practices included unlined evaporation ponds and lagoons where 483 chemicals including arsenic, polyaromatic hydrocarbons, dichlorobenzene and polychlorinated biphenyls (PCBs) are known or suspected to occur, with many detected in soil and groundwater [4]. LVW discharges directly into LVB, a popular fishing area within Lake Mead. 

**Figure 2:**
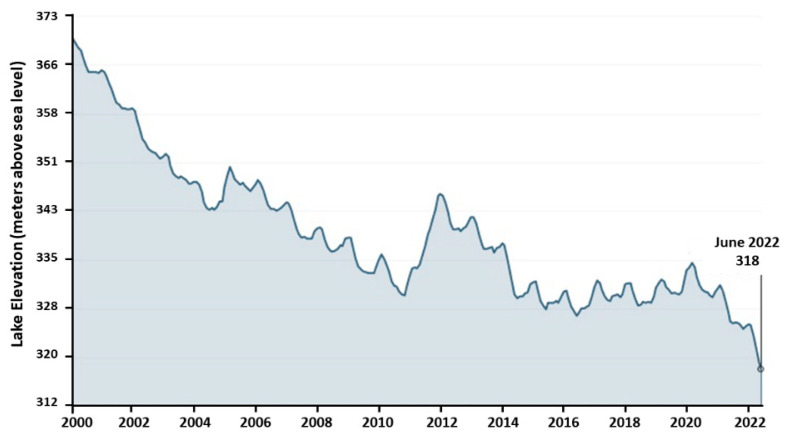
Monthly water levels at Hoover Dam for the past 22 years [11]. A long-term mega-drought in the Colorado River Basin has resulted in the lowest recorded water level in Lake Mead since it was created in 1937. Lower lake water levels provide less volume to dilute environmental organic contaminants discharged from Las Vegas Wash.

Water levels have continually dropped in Lake Mead more than 23 m over the past two decades (Figure 2) and reached the lowest recorded level in July 2022 [11]. The long-term mega-drought occurring over the last two decades in the southwestern United States, due to anthropogenic warming, is approaching a severity level of the highest magnitude in the last 12 centuries [12]. With mean yearly water losses from drought estimated at ≈1.4 Gigatons, the CRB was one of the 10 basins globally responsible for 70% of the total world-wide reservoir/lake water storage loss [13]. A continuing drought in the CRB will result in lower water levels in Lake Mead, with a recent study indicating that under a worst-case scenario, a drop in water surface elevation from 325 m (as of September 2023) to dead-pool of 274.3 m (water level where electric power cannot be generated) would increase wastewater discharge concentrations by 114% at the drinking water intake of the Southern Nevada Water Authority in Boulder Basin (Figure 1) [14]. 

For close to three decades, a multitude of studies of the LMNRA on EOCs and aquatic species, including Common Carp (*Cyprinus carpio*) (hereafter, carp), Largemouth Bass (*Micropterus salmoides*), Razorback Suckers (*Xyrauchen texanus*), and quagga mussels (*Dreissena* spp.), have documented effects on ecosystem and fish health and condition [3,15,16,17,18,19]. Effects in fish were primarily observed at LVB and LVW (Figure 1), including altered endocrine system function as well as atypical histological and reproductive biomarkers including sperm motility. Another focal site was WB—the only one of 14 sites in the entire CRB where gonadal intersex in carp has been documented (1 of 9 females), and male carp showed poor testicular development including inflammation, calcified deposits, pigmented cell aggregates (PgCA), edema and has some of the highest high polychlorinated biphenyl (PCB) levels (>0.8 µg/g wet weight [ww]) in whole body tissues in the CRB [20]. Poor gonadal condition was found in both male and female carp at WB in 2007/08 [4], and they had the highest PCB whole body levels out of four sites in LMNRA, with 17% of samples >0.8 µg/g ww. These documented effects are a concern for Razorback Suckers as this species is distributed all along the free-flowing Colorado River, from below Hoover Dam to Lake Mohave, including WB [21], and they are reproducing in Lake Mead’s Boulder Basin near the inflow of LVW (Figure 1) [22].

**Figure 3:**
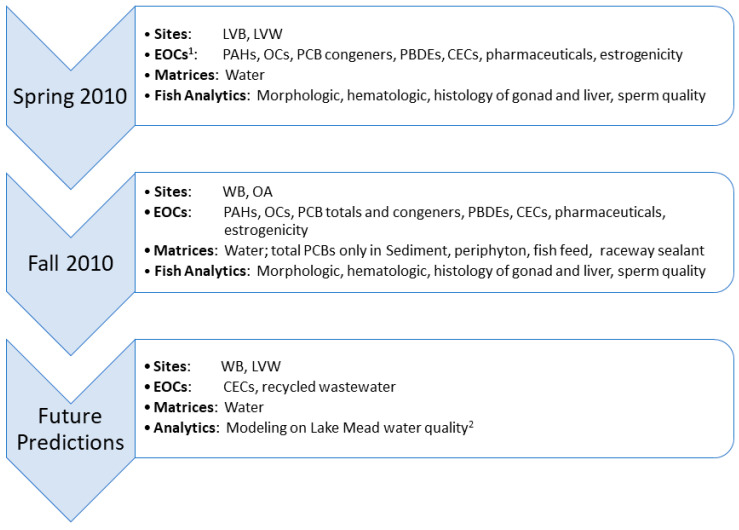
Overview and chronology of experimental activities presented in this manuscript focusing on male Common Carp (*Cyprinus carpio*), chemistry, and climate at LVB (Las Vegas Bay), LVW (Las Vegas Wash), WB (Willow Beach), OA (Overton Arm) in Lake Mead National Recreation Area, Nevada/Arizona, United States. EOCs ^1^ (environmental organic contaminants) detected in the water matrix, only; PAHs (polycyclic aromatic hydrocarbons); OCs (organochlorine pesticides); PCB (polychlorinated biphenyls); PBDE (polybrominated diphenyl ether flame retardants); CECs (contaminants of emerging concern). ^2^ Water quality models predictive of recycled water concentrations incorporating flow conditions, wastewater effluent, water and air temperatures [27,28].

Male carp were used as biomonitors of environmental health, including sperm quality biomarkers, as previous studies from LMNRA have shown their relative utility. They are bottom feeders where contaminated sediment can be ingested and reflect the general conditions of the site, they are collected in. The individual biomarkers chosen include standard measures used to assess general fish health and condition. The goals of this study were to compare fish health indicators in male carp at four LMNRA sites where a gradient of EOC exposure and a substantial range in water temperature regimes have been documented, and to assess how such relationships might be altered by changes in climate and regional drought. Specific objectives were to: (1) Assess potential PCB sources at WB and consider reasons why concentrations were highest at this site; (2) Determine the EOC gradient in water at WB, LVB, LVW and the reference site at Overton Arm (OA); (3) Investigate associations of EOCs and fish health indicators, including reproductive and histological biomarkers; and 4) Assess how changes in climate and regional drought in CRB could influence EOC exposures and consequent fish health/condition, including for the Razorback Sucker, as Lake Mead water levels continue to drop (See Figure 3 for chronology). The overall hypotheses for this study, based on multiple investigations by this team (refer to citations in above paragraph), are that contaminants are negatively influencing fish health, condition, and reproduction, and that projected further reductions in water levels will likely increase these effects.

## 2. Materials and Methods

### 2.1. Sample Collection and Chemical Analyses

#### 2.1.1. Passive Samplers

Passive samplers, including Semipermeable Membrane Device (SPMD) and Polar Organic Chemical Integrative Sampler (POCIS), were deployed in protective canisters at each site (Figure 1) using a concrete anchor and a buoy to suspend the samplers ~1 m above the lake bottom at depths ranging from 5 to 8 m. At each site, three SPMDs and ten POCIS, all contained in two deployment canisters, were deployed for ~1 month prior to biota being collected, 14 June–12 July 2010 (Spring; LVB, LVW), 14 October–17 November 2010 (Fall; OA, WB), after which they were retrieved and returned to the laboratory at Columbia Environmental Research Center, Columbia, MO. Methods for the processing and analysis of these samples have been described previously, with a list of individual analytes given in Appendix A (Table A1) [23,24,25]. Briefly, a single SPMD was analyzed for selected polycyclic aromatic hydrocarbons (PAHs), another SPMD was used for organochlorine pesticides (OCs), total polychlorinated biphenyls (tPCBs), five polybrominated diphenyl ether (PBDE) flame retardants, and the final SPMD was for selected wastewater (WW) chemicals [26] which were recovered using a hexane dialysis. Dialysates were treated to a series of sample fractionation and enrichment steps using size exclusion chromatography and adsorption chromatography. Final analyses for PAHs and WW were conducted by using gas chromatography/mass spectrometry (GC/MS), while OCs, tPCBs, and PBDEs were analyzed by using gas chromatography with electron capture detection.

A suite of chemicals related to WW and surface runoff were extracted from POCIS samples using a mixture of dichloromethane:methyl-*tert*-butyl ether (80:20 *v*:*v*) to extract samples for the identification of individual chemicals by GC/MS [24]. Methanol was used to recover select pharmaceuticals from the POCIS prior to analysis using liquid chromatography tandem mass spectrometry (LC/MS/MS). Extracts from two POCIS were composited for both the WW and pharmaceutical samples in order to increase the amount of chemical available in the final extract for analysis. Sample cleanup or fractionation was not done on POCIS extracts prior to analysis. Time-weighted average water concentrations of chemicals sampled by SPMDs and POCIS were determined by using first-order update models as previously described [29,30]. 

A separate, single POCIS from each site was extracted with methanol and screened for potential estrogenicity using the Yeast Estrogen Screen (YES assay). Estradiol equivalent factors (EEQ) for the water samples were calculated by measuring the effective concentration required to elicit a 50% response (EC50) in the exposed genetically modified yeast cells *(Saccharomyces cerevisiae*) for the 17β-estradiol (E2)–positive control and determining the percent of sample required to give an equivalent response [23]. The remaining POCIS were archived for future use. 

#### 2.1.2. Sediment, Periphyton, Fish Feed and Sealant Samples

At each of the five WB sites (inset Figure 1), sediment was collected using a petite ponar sampler suspended mid-channel from a boat. Attached benthic algae (periphyton) was sampled by scraping rocks in shallow water (<2 m depth) and sediment was rinsed off with native water, excess water shaken off, and placed in chemically clean glass jars. At the Willow Beach National Fish Hatchery (WBNFH), involved in the recovery of the endangered Razorback Sucker [31], additional samples of fish feed and raceway sealant consisting of rubber and commercial gap fillers were sampled to determine if these may be a secondary source of PCBs into WB. All sediment, periphyton, feed, and sealant samples were processed and analyzed for t PCBs using methods previously employed for fish tissue analyses in Lake Mead [4]. This involved accelerated solvent extraction, flash gel permeation and basic alumina fractionation/cleanup, and analysis by GC/MS [32].

### 2.2. Fish Collection and Sample Processing

Male carp used in this study were collected in July 2010 from LVB and LVW, and in November 2010 from OA and WB. Fish sampling procedures were as previously described [4]. Briefly, fish were captured with an electroshocking boat near the shoreline in water depths of up to 3 m. They were immobilized by pulsed DC current, captured with dipnets, and maintained in a live well until processed on shore. On each sampling date, fish from each site were collected on consecutive days over a 4-day period.

Blood was drawn from the caudal artery into heparinized syringes and centrifuged in capillary tubes on site for hematocrit determinations in duplicate (HematoSTAT II Microhematocrit, Separation Technology, Inc., Altamonte Spring, FL, USA), and the average was reported per fish. Fish were then immersed in a solution of tricaine methanesulfonate (MS-222, 1 g/L of lake water) until all movement ceased, then euthanized by a blunt blow to the head. Fork length (length), and body and testicular mass were measured. Animal handling was conducted in accordance with taxon-specific guidelines [33].

Testis and liver samples were collected in 2010 for analyses. Samples for histology were preserved in 10% buffered formalin, and those for sperm quality analyses were placed in calcium-free Hank’s balanced salt solution at pH 7.5 and 315 milliosmole/kg (HBSS) with 10% *v*:*v* 15,000 units of penicillin G sodium, and 5 mg streptomycin sulfate/mL (HBSS/PS). Testicular samples were shipped overnight on wet ice in HBSS/PS to the U.S. Geological Survey (USGS), Wetland and Aquatic Research Center, Lafayette, LA, USA for sperm quality analyses, and livers were transported to the USGS, Texas Cooperative Fish & Wildlife Research Unit, Lubbock, TX, USA for histological analysis. 

### 2.3. Histological Analyses

Gonadal and liver tissue samples were rinsed in 70% ethanol, processed, and embedded in paraffin following standard procedures [34]. Tissues were sectioned to 6 µm thickness and stained with hematoxylin and eosin, and liver samples were also stained with Periodic Acid Schiff (PAS) (Sigma-Aldrich, St. Louis, MO, USA) with or without prior α-amylase digestion (Sigma-Aldrich) for assessment of glycogen deposits. Digital images of histological specimens were taken with an Olympus digital camera (DP70; Tokyo, Japan). 

Testes were cut rostro-caudally into 10 equal segments, and a thinner sub-segment from each was processed for histological observations. For large testes, sub-segments were sectioned radially into 2–3 pieces prior to histological processing. Preparations from each segment were scanned for gross anomalies. In mid cross-sections of the testis, the relative area occupied by PgCA was determined using two diagonally aligned, adjacent images of the germinal area at 25×. These images were digitally analyzed with Image-Pro^®^ Express Software, v.4 (Media Cybernetics, Silver Spring, MD, USA) to measure the total area of the two images and the area occupied by PgCAs and reported as percent of total area. 

For livers, the percent area occupied by PgCA was evaluated from two digital images as described above. The accumulation of glycogen in hepatocytes was estimated based on analysis of two adjacent tissue sections, one directly stained with PAS and the other digested with α-amylase prior to PAS staining. Glycogen is depolymerized in the presence of α-amylase and washed out of the section but in undigested sections, is stained magenta. The difference in intensity of the magenta coloration between the undigested and digested sections was ranked as: 1 = minimal, 2 = moderate and 3 = high. 

### 2.4. Sperm Quality Analyses

Several tests assessing sperm quality were performed and described below. More details and data are available in [35,36]. Milt from testes arriving after overnight shipment for morning arrival was obtained from the collecting duct at the posterior testis end.

#### 2.4.1. Apoptosis and Viability

Milt (1 µL) was added to 99 µL of cold (8 °C) annexin- binding buffer (BB) [37] and centrifuged at 300 rcf for 5 min, then resuspended in 100 µL BB at 1 × 10^6^ cells/mL. Cells were stained with 5 µL annexin V (cat#A13199, fluorescein conjugate; Life Technologies/ThermoFisher Scientific, Carlsbad, CA, USA) and counterstained with propidium iodide (PI; 2.5 µL of a 2.4 mM stock solution). After incubation in the dark for 15 min, 400 µL of BB was added, and then staining controls and duplicate experimental samples were analyzed by flow cytometry (FCM) with 10,000 events per tube generated in biexponential plots. Data were analyzed with the curly quadrant tool (FlowJo™ Software, v.10.7.0, Ashland, OR, USA, Becton Dickinson and Company). Percentages of total apoptotic, total live, and live apoptotic cells were calculated after initial gating of the singlet population and out-gating cells on cytogram edges.

#### 2.4.2. Mitochondrial Membrane Potential

Cells at 1 × 10^6^ cells/mL were incubated with Rhodamine 123 (5 µL of 0.13 µM solution; Life Technologies) and PI (2.5 µL of a 56 µM solution) and analyzed in duplicate at 10K events each by FCM [9]. Data were later analyzed with FlowJo.

#### 2.4.3. DNA Integrity

Milt at 2 × 10^6^ cells/mL was stained with an equal volume of PI (25 mg/mL) solution for 30 min at 24 °C in the dark [38] and nuclei were analyzed by FCM at fewer than 300/s with 10K nuclei analyzed per sample in duplicate. Data were analyzed with FlowJo for DNA integrity by nuclei outside the main population (NOMP) [37], by percent of haploid and cells with more than haploid DNA content [9,39], and for coefficient of variation by using model 1DAOn_DSD with Modfit LT version 3.3.11 (Verity Software House, Topsham, ME, USA).

#### 2.4.4. Spermatogenic Staging

To assess relative numbers of haploid cells and those in earlier stages of spermatogenic development, a representative testicular cross section of approximately 0.2 g was minced for 1 min in 200 µL HBSS then fixed in 800 µL of 4% paraformaldehyde in phosphate buffered saline (Invitrogen). Days later, fixed testicular cells were diluted to 2 M/mL, filtered through 30-µm nylon mesh (Component Supply, Sparta, TN, USA), stained with PI solution, and incubated at 24 °C for 30 min. In duplicate, 10,000 events were collected by FCM at 300 cells/s or slower, with initial gating on fluorescence 2 area (FL2-A) vs. width, followed by determining the percentage of haploid nuclei out of the total events as displayed with FL2-A histograms [9,38].

#### 2.4.5. Sperm Motility

Aliquots of milt (0.25 µL) were activated with tap water (25 µL) and motilities were measured by computer assisted sperm motion analysis assessed in a chambered slide (Leja 20 catalog number SC20-010040-B, Leja Products, Nieuw-Vennep, The Netherlands). Cells were viewed with phase microscopy (Olympus BX41, Olympus America, Inc, Center Valley, PA, USA) at 200 times total magnification. Data from approximately 500 cells per sample were electronically captured at 60 frames/s and analyzed with SpermVision, v. 3.0 (Minitube of America, Verona, WI, USA). Percent of total- and progressively motile cells were reported.

#### 2.4.6. Sperm Morphology

One µL of milt was diluted with 99 µL HBSS, negatively stained, then viewed at 600× magnification (Olympus BX41) [37]. At least 500 cells per slide, with duplicate slides per individual, were scored for the two most observed abnormalities (macrocephaly and cytoplasmic droplets).

#### 2.4.7. Sperm Counts

Starting with 1 µL of milt fixed in 99 µL of 4% paraformaldehyde (cat. no. FB002; ThermoFisher Scientific, Waltham, MA, USA) having been stored at 8 °C, an aliquot of 0.25 µL was diluted into 500 µL HBSS and analyzed by FCM with Bacteria Counting Kit (B-7277, Molecular Probes, Eugene, OR, USA) [9]. Duplicate counts of 20,000 events were performed on replicates and recounted if replicates varied by more than 15 percent of the standard deviation to generate counts per mL milt.

#### 2.4.8. Adenosine Triphosphate (ATP) Content 

The quantity of ATP per 1 M cells was determined by using an ATP-binding luciferase assay (ViaLight Plus Kit, cat. no. LTo7-221, Lot. No. 1309016; Lonza Rockland, Inc., Rockland, ME, USA) according to manufacturer’s instructions and data collected with a TECAN GENios plate reader (Mannedorf, Switzerland). Standard curves were generated each day samples were run. For each sample, 50 µL from a 2 µL live milt in 198 µL initial dilution (also counted later by FCM from fixed suspensions that were processed and luminescence intensities (relative light units; RLUs) were measured and used to calculate ATP concentration per number of cells [36].

### 2.5. Data Processing and Analyses

Gonadosomatic index (GSI) was determined according to the formula GSI = (gonad mass/body weight) × 100. All biological data were analyzed by Principal Component Analysis (PCA), a multivariate analytical procedure that reduces datasets of multiple variables to a lower number of orthogonal variables (principal components, PCs), and is useful for exploring patterns [40]. Thus, PCA was applied to fish datasets for March 2007, July 2010, and November 2010. Statistical analyses for tPCBs in whole of body carp were performed using data from Patiño et al. (2015) and log transformed to meet the assumptions of normality, with α = 0.05. One-way ANOVA was used for determining statistically significant biological variables between LVB and LVW, and OA and WB, according to seasons sampled as gonadal development and reproductive processes follow seasonal changes [16].

Except for histological measurements, all variables were transformed to normal scores prior to PCA using the Blom method in SAS Proc Rank [41], thereby achieving or improving Gaussian data distributions. Testis and liver PgCA and glycogen rank, not normally distributed, were transformed for PCA using monotonic spline (for quantitative variables) or monotonic/untie (for ordinal variables) options in SAS Proc Prinqual [41]. The prinqual procedure optimizes the properties of the correlation matrix so that PCA can be applied to qualitative, quantitative, or mixed datasets. The identity transformation was applied to the variables previously transformed to normal ranks to keep their values unchanged.

The PCA graphs were generated from the PRINQUAL output using PROC TEMPLATE & SGRENDER in SAS [41]. The number of principal components selected for interpretation was determined by Parallel Analysis which retains only those components with eigenvalues greater than the 95th percentile of random, Monte Carlo-simulated eigenvalues [42,43]. Principal component biplots were generated to graphically explore potential patterns in data distributions. Component scores were used for a rigorous assessment of differences among sampling sites within a selected component using one-way ANOVA and Tukey’s multiple comparison tests (March 2007) or *t*-tests (July and November 2010; two-sided, α = 0.05). 

### 2.6. Lake Mead Water Quality Model

A full mathematical three-dimensional hydrodynamic and water quality model for Lake Mead was used to simulate probable future scenarios of lowering water levels due to climate change and regional drought. This model is implemented in Aquatic Ecosystem Model 3D (AEM3D), that approximates quantities of interest by solving the Reynolds-averaged Navier-Stokes equations with a turbulent eddy closure and solves for hydrodynamics and water quality [44]. The model has been maintained, calibrated, and updated by SNWA for over 20 years [45]. The model inflows are the Colorado River which accounts for 97% of the inflow volume, with smaller contributions from the Virgin and Muddy Rivers and LVW. Most water is released through Hoover Dam, with minor withdrawals from Southern Nevada Water Authority’s (SNWA) drinking water intake plus model-computed losses to evapotranspiration [46].

Validation of the LM3 (Lakes Mead and Mohave Model) in Lake Mead was performed by sampling sucralose (log Kow 1.67) in the treated effluent (LVW) and then comparing that concentration to the concentration at the drinking water intake in southwestern Lake Mead, Boulder Basin (Figure 1), that agreed with model results of 1.3% recycled water contribution (RWC) [14]. This approach was further validated with data in this study using methyl triclosan (log Kow 4.76), a degradant of an antimicrobial commonly used in soaps, deodorants, and other personal care products where water concentrations in LVW of 15.3 ng/L were compared to 0.15 ng/L at WB station three [26] giving an RWC of 1% that is relatively close to the LM3 model results of 1.3% RWC using the much more soluble sucralose. This gives some confidence that the LM3 model results using sucralose work reasonably well as a surrogate for more hydrophobic EOCs.

The model was run in 2023 for two sites, Hoover Dam and WB, as the two 2010 passive sampling sites in Lake Mead were above the water level. The LM3 was used to approximate dilution of a conservative tracer (sucralose, an artificial sweetener) for previous (2010) and more recent (2022) flow conditions [27]. Dilutions of highly treated WW effluent from LVW reported as RWC in Lake Mead from 2010 and 2022 [27] were compared to determine how measurements may change under current 2023 lake conditions. Hydrodynamic models are not available for LVW; however, mean flow rates were compared for the 12 years between 2010 and 2022 to determine the difference in WW loading into Lake Mead.

For predicting how water temperatures would change at WB, 4.8 °C was added to air temperature [27,28,47]. This is in accordance with RCP 8.5/late century warming projected for Clark County, NV. Wilcoxon rank sum test (MATLAB: rank sum) was used to compare RWC during historic and projected climate scenarios.

## 3. Results and Discussion

### 3.1. PCBs at Willow Beach

The sum of all PCB congeners (tPCBs) for five sites along an 8 km stretch in the Colorado River above and below WB (Figure 1) detected in water, sediment, periphyton, as well as in fish feed, fish tank caulking and gap-filler used at the WBNFH are presented in Table 1. These samples were taken based on PCA results from a 2007/08 study where tPCBs in male carp whole bodies was a variable, and WB clearly had higher concentrations than the other three sites in LMNRA [4]. The overall mean tPCBs from WB from that study was 408 ng/g ww in male carp (n = 54), being the highest of all sites. A previous study [20] also showed WB male carp contained high PCB levels (870 ng/g ww), being the second highest level among the 14 sites in the CRB. Thus, the question surfaced as to why did carp at WB have such high PCBs levels compared to the other three LMNRA sites? 

Leakage from old electric transformers at Hoover Dam could be a potential source [4]. The two upstream sites above WB showed possible PCB sources as they were the only detections in sediment of five sites (Table 1). However, periphyton values indicated PCBs sources at WB with the highest tPCB values of 4.8 ng/g dry weight (dw), and then at the farthest downstream site of 5.9 ng/g dw (Table 1). These values are at least an order of magnitude lower than tPCBs levels of 40–325 ng/g dw in periphyton from Lake Mead [7]. A potential route of PCB exposure to WB carp was observed from many fish feeding on the remnants of fish feed at the WBNFH outfall that had PCB concentrations ranging from 3.4–9.1 ng/g dw (Table 1). These PCB concentrations are comparable to the mean of 6.85 ng/g dw found in fish feed samples from the U.S. [48]. Other sources of PCBs at WBNFH included caulk and gap-filler, showing an order of magnitude higher levels (Table 1) than fish food. In this study, because no PCBs were detected in water (method detection limit 460 pg/L) at any of the five WB sites, there does not appear to be a large PCB source in this stretch of the river from upstream sources.

**Figure 4:**
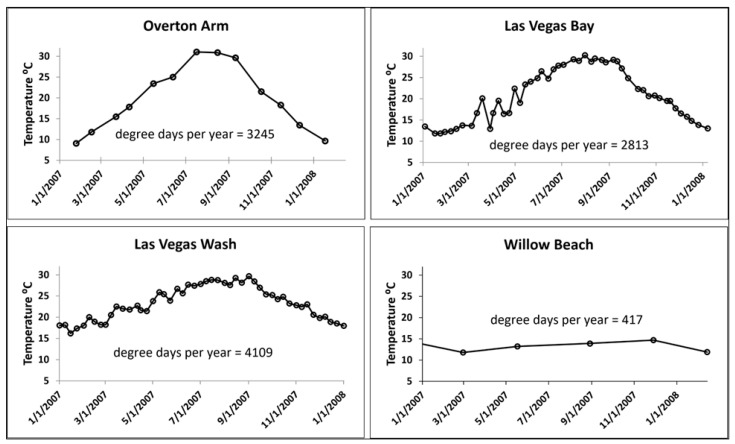
Water temperatures at four sampling sites in Lake Mead National Recreation Area NV and AZ, US where common carp (*Cypinus carpio*) were sampled over a one-year period to analyze accumulation of environmental organic contaminants. Degree days over that period were calculated by summing all the temperatures above 12 °C, which initiates growth in common carp [53]. Willow Beach had substantially lower degree days (up to 10 times), indicating slower growth and longer lifespans.

A possible explanation of higher PCBs in WB carp despite low PCBs in water and fish feed remnants in the hatchery outfall is male carp have the highest mean age of 44 years (up to 54 years) in the CRB [20]. There are several studies that show EOCs (including PCBs) accumulate with fish age [49,50,51]. The mean age of WB male carp was 10 years older than carp at any other site in the CRB [20], and where year-round water temperatures are between 12–14 °C, being considerably lower than the other three sites in LMNRA (Figure 4). Cold water reduces metabolism and respiration, thus enhancing EOC accumulation [51]. Moreover, carp lifespan was shown to be longer in colder climates (therefore colder water temperatures) in North America [52]. The low threshold for initiation of carp growth, 12 °C [53] significantly reduced the degree days for growth at WB compared to the other three sites between 670–980% (Figure 4) and could explain the long-lived fish. In spite of WB male carp median PCBs levels of 207 ng/g ww being comparable to the median value of 228 ng/g ww from a reference site on the Hudson River, NY [54], the very long chronic exposures (up to 54 years) at WB are associated with reduced reproductive potential (Section 3.4) and testicular cancer.

### 3.2. Environmental Organic Contaminants in Water

All EOC results in water can be found in Alvarez and Echols (2024). Of the 130 EOCs analyzed, including metabolites and degradants, 72% were detected in passive sampling extracts in 2010: 18% (24) in OA; 38% (49) in WB; 50% (65) LVB; and 55% (72) LVW (Table 2). The site gradient for the number of EOCs detected is very clear. OA < WB < LVB < LVW (Table 2). Total EOC sums were ≈3 to 19 Xs higher in LVW compared to other sites and LVB was ≈5 Xs higher than OA and WB (Table 2). Three years after this study in 2013 and 2014 [7], results from passive sampling performed at LVB and OA showed lower values for both the number of compounds detected and the total EOC group sums detected in this 2010 study. The lower levels of EOCs in 2013/14 may be in part due to the higher populations of quagga mussels accidentally introduced to Lake Mead in 2007/08 with the population quickly expanding over time [7]. Quagga mussels are efficient filter feeders that consume plankton and organic detritus and will therefore accumulate and concentrate contaminants directly from the water column and particulate matter [55]. Hence, substantial quagga mussel populations can remove significant EOC mass from the water column through filter feeding. In 2012, the population number in Lake Mead was estimated to be 1.5 × 10^12^ [6], where 3.3 kg of EOCs were estimated to have been removed from the water column by quagga mussels compared with 31.3 kg of EOCs in the entire lake water column [7].

With a substantial number of EOCs detected that are relevant for water quality criteria, screening values and benchmarks were used to assess potential toxicity and effects on aquatic biota. It is interesting that none of the legacy EOCs exceeded any chronic water quality criteria and were substantially lower even at LVW, the most contaminated site (Table 3). Mirex, a legacy organochlorine pesticide that was banned in 1976, was detected at two sites, LVW (0.0013 ng/L) and at WB (0.011 ng/L). This WB site, number three (Figure 1), is where the WBNFH has an outfall, suggesting a potential Mirex source in that area. Although the Mirex concentrations at these two sites were at least two orders of magnitude under the aquatic life criteria of 1.0 ng/L (Table 3), the detections indicate historic sources.

In contrast to legacy contaminants, a newer group of EOCs, contaminants of emerging concern (CEC), include tens of thousands of very diverse compounds where many are ubiquitous in surface waters, are a source of growing concern [56]. Of the four CEC detected from POCIS (Table 4), each of the four sites showed concentrations that exceeded the ecological screening values that are used for assessing relative hazard to freshwater fish from chronic aqueous exposures in surface water [56]. At 3600 ng/L, Galaxolide (HHCB), a polycyclic musk widely used in fragrances, substantially exceeded both the low comprehensive screening value (LCSV) of 64.9 ng/L and the low population relevant screening value (LPRSV) of 910 ng/L at LVW (Table 4). Although the Galaxolide concentration indicated a low risk to aquatic biota in LVW, it is well below the high comprehensive screening value of 21,300 and high population relevant screening value (HPRSV) of 60,200 ng/L [56]. Concentrations of two other CECs, N,N-diethyltolumide (DEET), a common insect repellant, exceeded the LPRSV of 1.3 ng/L ng/L at all four sites indicating it is widely used (Table 4). The DEET concentration of 390 ng/L at LVW also exceeded the LCSV of 23.6 ng/L by more than an order of magnitude, but the values were well below the HPRSV of 7 × 10^5^ ng/L (Table 4). This concentration was higher than 51 wastewater effluents out of 58 reported Worldwide [57]. Another CEC, triclosan is a commonly used antimicrobial in deodorants and toothpastes and it exceeded both high and low screening levels at WB and LVW (Table 4). It has a number of effects in aquatic ecosystems including reduced growth in algae and both reproduction and development in fish [7]. 

Of the nine pharmaceuticals analyzed within water samples, only four (44%) were detected, with all four found at LVW, three at LVB, and none at WB or OA [26]. The concentrations at LVB and LVW were generally much lower than those reported in U.S. streams [58], and the calculated risk quotient indicated they posed minimal ecological risk (Table A2). A concentration gradient was clear regarding the pharmaceuticals from their sewage treatment plant source in LVW, with the highest concentrations lowered by 11–50% in receiving waters at LVB through dilution, sorption to sediment, or degradation, and then no detections occurred further downstream at WB [26]. This gradient can be explained by the very short half-lives of pharmaceuticals when they are photodegraded in water, such as 24.9 h for azithromycin and 17.7 h for clindamycin [59].

There was a clear gradient of the 34 PAHs analyzed in water with only 1 detected in OA, 12 in WB, 15 in LVB and 17 in LVW (Table 3). The total sums in pg/L also had the same gradient as the number of detections, OA 35 pg/L, WB 1223 pg/L, LVB 2053 pg/L, and LVW 2532 pg /L (Table 3). Assessing the ecological significance of the 16 PAHs of most concern was done by using EPA benchmarks for screening expressed as Toxicity Equivalent Factor (TEF) [60] in pg/L Benzo[a]pyrene (BaP). These TEFs showed a gradient similar to the one using water concentrations for 34 PAHs, OA 0 pg/L, WB 5.25 pg/L, LVB 9.4 pg/L, and LVW 14.4 pg/L (Table 5). All TEFs were at least three orders of magnitude below the benchmark for ecological protection of 15,000 pg/L [61]. The concentrations of five known carcinogenic PAHs were similar among the three sites at which they were detected, three from LVB, and four from both LVW and WB. The most carcinogenic PAH, B*a*P was only detected at LVB [26].

**Table 3:** Water concentration of environmental organic compounds (EOC) at four study sites in Lake Mead National Recreation Area and their chronic aquatic life criteria. Values (ng/L) were estimated by using semipermeable membrane device samplers deployed over a ~30-day period prior to sampling biota in 2010.

EOC Group		Sampling Sites			
Organochlorine Pesticides	Chronic Aquatic Life Criteria ^1^	Overton Arm	Willow Beach ^2^	Las Vegas Bay ^3^	Las Vegas Wash
cis-chlordane	4.3	ND ^4^	0.0038	0.048	0.058
trans-chlordane	4.3	ND	ND	0.026	0.049
p,p’ DDT	1	ND	0.0077	ND	0.12
Dieldrin	56	0.063	0.018	0.064	0.085
Endrin	36	0.016	0.0099	0.02	ND
Lindane	950	ND	ND	0.04	0.14
Chlorpyrifos	41	0.11	0.029	0.065	0.13
Methoxychlor	30	ND	ND	ND	0.029
Mirex	1	ND	0.011	ND	0.0013
Endosulfan	56	ND	ND	0.15	1.8
Heptachlor epoxide	3.8	0.0069	0.023	0.046	ND
**Polychlorinated Biphenyls**					
Total PCBs	14	ND	ND	0.61	0.42
**Polycyclic Aromatic Hydrocarbons**					
Acenaphthalene	23,000	ND	ND	ND	0.32
Anthracene	1.3	ND	ND	ND	ND
Benz [a] anthracene	27	ND	0.037	0.02	0.37
Benzo [a] pyrene	27	ND	ND	0.013	ND
Fluoranthene	6160	ND	0.35	0.44	0.5
Phenanthrene	3230	ND	ND	ND	0.85

^1^ [58]. ^2^ Highest detection of five sampling sites within Willow Beach area. ^3^ Highest detection of surface or bottom sample. ^4^ ND, not detected.

**Table 4:** Screening values for the contaminants of emerging concern (CEC) detected at four study sites in Lake Mead National Recreation Area for assessing relative hazard to freshwater fish from chronic exposures.

Category	CEC	Screening Value ^1^		Overton	Willow	Las Vegas	Las Vegas
		LC ^2^	LPR ^3^	Arm	Beach ^4^	Bay ^5^	Wash
Fragrances	Galaxolide (HHCB)	64.9	910	ND	ND	**140** ^6^	***3600*** ^6^
Insect repellants	N,N-diethyltolumide	23.6	1.3	**2.2**	**2.2**	**19**	***390***
Antibacterials	Triclosan	2.5	2.9	ND	***5.2***	2.3	***8.3***
Flame retardants	Tris-2(butoxyethyl)	480	1670	ND	ND	ND	160
	phosphate						

^1^ Screening Values in ng/L [56]. ^2^ Low Comprehensive Screening Values (LCSV) developed using all available effects information; values below LCSV predict negligible hazards to fish. ^3^ Low Population Relevant Screening Values (LPRSV) developed using conventional population-relevant effect information; values below LPRSV predict negligible hazards to fish. ^4^ Highest value of five Willow Beach sites (See inset of Figure 1). ^5^ Highest value at surface or bottom. ^6^ **Bold** exceeds one SV; ***Bold*** exceeds both.

**Table 5:** Toxicity Equivalency Factors (TEF) ^1^ of Polycyclic Aromatic Hydrocarbons (PAH) detected in water sampled from sites ^2^ below Hoover Dam in 2010.

		Willow	Las Vegas	Las Vegas
PAH	TEF	Beach	Bay	Wash
Acenaphthylene	0	ND	ND	0.32
Fluorene	0	ND	0.035	0.11
Phenanthrene	0	ND	ND	0.85
Fluoranthene	0	0	0.308	0.335
Pyrene	0	0.266	0.265	0.36
**Benzo (a) anthracene ^3^**	0.1	2.58	1.8	3.7
**Chyrsene**	0.01	0.67	1.1	1.9
**Benzo(b)fluoranthene**	0.1	0.84	ND	4.7
**Benzo(k)fluoranthene**	0.1	1.16	ND	2
**Benzo(a)pyrene (BaP)**	1	ND	6.5	ND
Benzo(a,h,i)perylene	0.01	ND	0.39	ND
Sum per site		5.82	10.94	14.44

^1^ Toxicity Equivalent (pg B[a]P eq/L); TEF method evaluates structurally related compounds sharing a common mechanism of action compared to a single compound [58]. ^2^ Only perylene was detected at Overton Arm at a level not generating a TEF. ^3^ Bold compounds are known carcinogens (USEPA 1993: https://rais.ornl.gov/documents/600R93089.pdf; accessed 11 September 24).

### 3.3. Estrogenic Activity

Results from the YES assay showed estrogenicity reported as estradiol equivalents (EEQ) at only two sites, OA (0.39 ng/L) and WB (3.1 ng/L) site three near the WBNFH (Figure 1) and none at LVB. The sample at LVW was toxic to the yeast used in the assay across multiple dilutions indicating very high toxicity and therefore no EEQs were able to be generated. Estrogenicity can also be estimated by using estrogen receptor agonist activity (ERAA) [62] for the 15 estrogenic EOCs detected. These results (Table 6) proved useful and show a clear gradient, LVW > LVB > WB > OA, with an order of magnitude difference among sites.

One interesting YES result was that the highest EEQ (3.1 ng/L) was detected at WB site three with no detections upstream or downstream. However, the ERAA value for this site of 0.0118 ng/L (Table 6) was only a fraction of the YES result, indicating other estrogenic compounds were present, but were not specifically analyzed at this site. Thus, the likely source of estrogenicity was from the WBNFH where the estrogenic effects were detected in water near the outfall. Estrogenic chemicals, mostly phytoestrogens, and estrogenic activity between 0.12–6.2 ng/L/g in fish food were found in 12 of 15 commercial fish feeds [63]. Considering the large amounts of feed observed in the WBNFH outfall, it is suspected fish feed may be the primary source of estrogenicity measured in the water at this site, which is well above the 0.1–0.4ng/L long-term EEQ environmentally safe level [64]. 

The estrogenic activity results determined with ERAA showed a gradient among sites. However, these are underestimates because every estrogenic chemical occurring at each site was not included, such as the sex steroid hormone 17β estradiol (E_2_). Because there are no YES results from LVW, the 5.36 ng/L, ERAA is helpful in assessing potential effects on fish health and reproduction at that site. Past results from LVW [65] showed E_2_ as high as 2.7 ng/L (E_2_ was not measured in this study) and when combined with the calculated ERAA (≈8.1 ng/L), this value provided a better estimate of estrogenicity at LVW. Generally, an estrogenicity of <1.0 ng/L is considered a reference value, and values of 1.0–3.0 ng/L are moderate [66]. Estrogenic values have a wide range in water with values as high as 242 ng/L in the heavily farmed Imperial Valley to non-detects (<0.15 ng/L) in Sierra Nevada foothills of California, United States [67]. Safe environmental EEQ levels for aquatic biota were developed by using a variety of in vitro assays [64]. The long-term chronic value (exposure for >60 days) of 0.1–0.3 ng/L was well exceeded in both LVW and WB. 

Some studies have been performed along gradients of environmental contaminants gradients to test the hypothesis that reproduction in male fish is associated with exposure to EOCs. One such study in the lower Columbia River assessed reproductive and endocrine parameters in male resident Largescale Suckers (*Catostomus macrocheilus*) [37]. Sperm quality parameters were significantly lower and vitellogenin (Vtg), a hormone used in female fish for egg production, was higher in males at the site where liver contaminants in fish and EEQs in water were highest. Correlations were found among specific contaminants and reproductive or endocrine parameters: total concentration of PBDEs were negatively correlated with sperm motility, PCB-206 and BDE-154 were positively correlated with DNA fragmentation, and thyroxine (T4) [37]. In the CRB, sperm viability of Lake Mohave carp (79%), just below WB, was significantly lower than that from carp at Lake Havasu (95%) [68]. Also, gamete quality, endocrine, and reproductive data were collected among LMNRA sub-basins over 7 years (1999–2006); diminished biomarker effects were noted in 2006, and sub-basin differences were indicated by the irregular occurrences of contaminants and by several associations among chemicals (e.g., PCBs, hexachlorobenzene, galaxolide, and methyl triclosan) and biomarkers (e.g., T4, sperm motility and DNA fragmentation) [18,69].

In this study, the site with the highest estimated estrogenicity, LVW (≈8.06 ng/L), was well below the lowest estrogenic values shown to impact sperm motility in fish (600 ng/L), but was not far from the 19 ng/L associated with Vtg induction in goldfish [70,71]. The highest concentration in LMNRA of the weakly estrogenic DEHP of 0.72 µg/L at WB is close to the 1.0 µg/L that decreased sperm production, motility, and velocity in goldfish [72]. In spite of no measured individual estrogenic compounds being above concentrations that cause effects in lab studies, EEQs were well above long-term environmentally safe levels at LVW, where fish showed significantly lower sperm motility (*p* < 0.05), significantly more DNA fragmentation (*p* < 0.0001), and significantly more sperm cell forms indicative of not being reproductively mature (*p* < 0.001) compared to LVB (Table 6). These observations suggest that other environmental stressors, in addition to estrogenic compounds, can influence the reproductive potential of fish [18,37,68]. The high concentration of HHCB above water quality criteria in LVW could be a factor for the lower sperm quality there, compared to LVB.

### 3.4. Biological Variables

**Figure 5:**
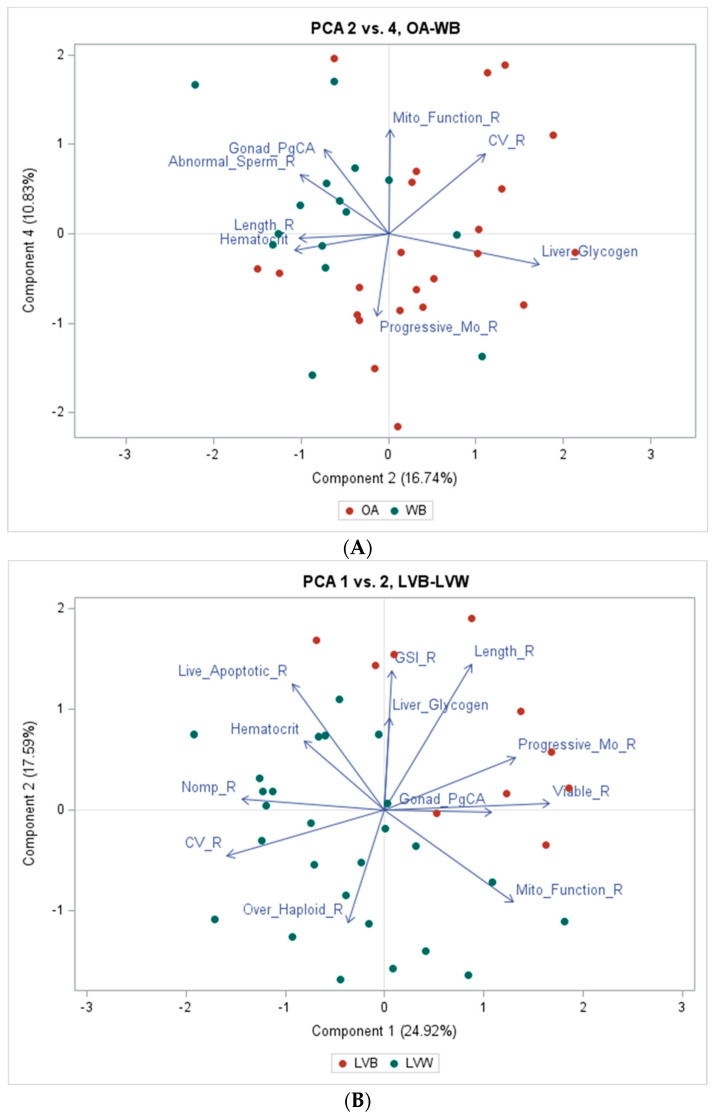
(**A**) Fish from Overton Arm (OA) and Willow Beach (WB) collected in November 2010, and (**B**) from Las Vegas Wash (LVW) and Las Vegas Bay (LVB) in July 2010. In the OA-WB plot, OA data appeared evenly distributed among all quadrants, except the upper left, which was occupied mostly by WB data. Principal Component vectors show lower liver glycogen, higher incidence of testicular pigmented cell aggregates and more abnormal sperm compared to OA indicating exposure to environmental organic contaminants (EOCs). (**B**) In the LVW-LVB plot, LVW data appeared evenly distributed in all quadrants except the upper right, which was exclusively occupied by LVB data. PC vectors show lower progressive sperm motility, higher % haploid sperm and higher DNA fragmentation in LVW compared to LVB indicating exposure to EOCs. Ranked value analysis is represented by “R.” Pigmented cell aggregates is “PgCA”. Gonadosomatic index is “GSI”. Mitochondrial is “Mito”.

A summary of biological variables is shown in Table 7 for all four sites with statistical differences noted between LVW and LVB samples collected in July 2010 and OA and WB samples collected in November 2010. A multivariate analysis using PCA was performed to assess the most important biological variables separating sites within each sampling period (Figure 5). From the principal component (PC) retained in the analysis, the only PC combination that indicated a graphical pattern in the separation of multivariate fish data at OA from WB was that of PC2 and PC4; it accounted for only 27.6 percent of data variability, indicating that unknown physiological and morphometric variables (other than those included in this PCA) may be necessary to fully describe site differences among fishes.

Data for most OA fish were found to the right of PC4, suggesting a unidimensional association with higher values of PC2 (Figure 5A). Thus, based on the orientation of vector variables that met the significance criteria for interpretation (component loadings > 0.4), most OA fish showed relatively high content of liver glycogen compared to WB (Figure 5A), which was also statistically higher (*p* < 0.05) than WB that had higher EOC concentrations (Table 2). A common target tissue for contaminants in vertebrates is the liver, especially for ingested chemicals [73]. PCB accumulation in fish from WB was over an order of magnitude higher than fish from OA (Figure 4). Changes in hepatic glycogen are a common response to toxic exposures or diet unbalance [74]. Glycogen, for example, may be depleted in the liver under stressful conditions due to the rise of serum glucose [75,76,77]. In this study, male carp from WB had lower glycogen levels than fish from OA, supporting the hypothesis that fish from WB may be exposed to environmental stressors that were affecting overall fish health. 

**Figure 6:**
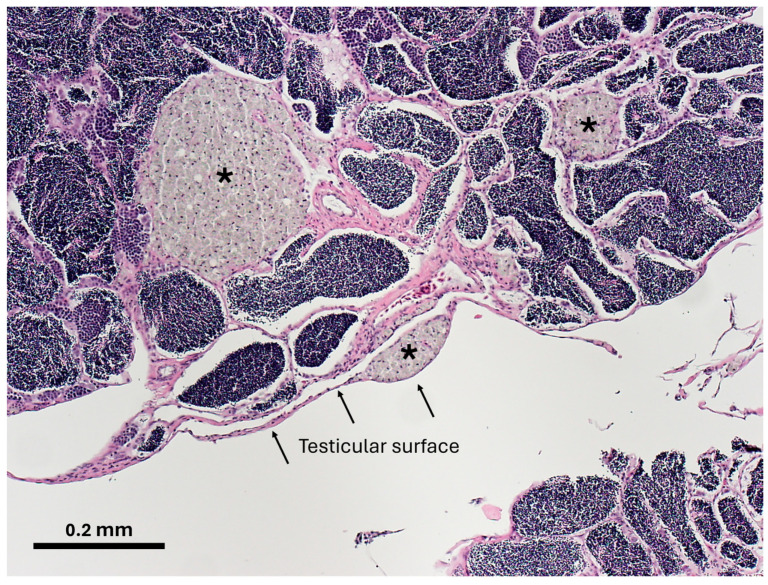
Photomicrograph of pigmented cell aggregates (asterisks) in the testis of a male common carp collected from Willow Beach. The cell aggregates take on a yellow–brown coloration when stained with hematoxylin and eosin, which was previously shown to represent ceroid–lipofuscin deposition [16]. When present, pigmented cell aggregates could be found throughout the testes, including near their surface.

Conversely, data for most WB fish were found in the upper-left quadrant of the plot (Figure 5A), suggesting that they were simultaneously associated with increasing and decreasing values of PC4 and PC2, respectively—namely, most WB fish had higher incidences of abnormal sperm (Figure 5) and testicular PgCA (Figure 6). The considerably high prevalence of testicular PgCA, almost an order of magnitude higher than fish from OA (and almost double what was observed in LVB and LVW) was statistically significant (Table 6). These results are supported by another study [20], whereby carp at WB and South Cove, upstream of Lake Mead, showed the highest percentage and area of PgCA in spleen not only in the CRB, but in the nationwide USGS Large River Monitoring Network Program [20]. High incidences of PgCA in fish tissues have been used as an index of environmental exposure to contaminants in aquatic environments [78,79,80]; moreover, it has been shown that testicular PgCAs may affect steroidogenesis [81] and the regulation of spermatogonial proliferation [82].

The combination of PC1 and PC2 in the PCA for LVW and LVB yielded the clearest pattern of multivariate data separation. With a few exceptions, data from most LVB fish were in the upper-right quadrant of the plot while data from LVW fish were distributed across the other three quadrants (Figure 5B). This pattern of data distribution suggests that most LVB fish are simultaneously associated with relatively high values of length and progressive sperm motility (Figure 5B). Length in LVB fish was significantly greater (*p* < 0.05) than fish from LVW, most likely due to much smaller available stream habitat in LVW compared with a much larger lacustrine habitat in LVB. Progressive sperm motility was 31% higher in LVB than LVW (*p* < 0.05), with total EOCs being 73% lower in LVB compared to LVW (Table 2). The distribution of data from LVW fish in the PCA was best described by fish length, percentage of sperm being less reproductively mature, and large coefficient of variation (CV) in DNA indicating less DNA integrity. For example, a fish with lower length (associated with negative values on PC2 and thus lower values of fish length), higher levels of immature sperm forms and higher DNA fragmentation (CV) would be a fish from LVW (Figure 5B). PC1 and PC2 accounted for 42.5% of data variability. 

Fish from LVW had significantly (*p* < 0.001) more reproductively immature sperm forms (57%) than LVB fish (Table 7). Spermatogenesis involves mitosis, meiosis, and cellular differentiation in the production of mature haploid sperm [37]. Genotoxic effects from contaminants can increase the number of diploid spermatids in rodents due to failure of meiotic chromosomes to separate [83]. Results from a field study in the Columbia River, WA, showed higher percentages of immature sperm stages in Largescale Suckers from sites with higher contaminants than the reference site indicating potential effects on reproduction [37]. Similar results in another study in the Imperial Valley, CA, showed a higher percent of immature sperm forms in Western Mosquitofish (*Gambusia affinis*) at sites contaminated with organochlorine insecticides compared to fish from a reference site [77].

Fragmentation of nuclear DNA that alters integrity in fish chromatin has been related to effects on individuals as well as populations [37], and can be caused by exposure to aromatic hydrocarbons in English Sole (*Parophrys vetulus*) [84]. DNA fragmentation as measured by coefficient of variation (CV), was a significant variable in the PCA analysis, separating LVW from LVB (Figure 5) and significantly higher (*p* = 0.0001) in LVW, 4.9% compared to LVB, 2.5% (Table 7). LVW showed both 19% higher PAH concentrations in water (Table 2) and 19% higher PAH TEFs (Table 5), and lower sperm DNA integrity at the more contaminated site.

GSIs were 36% lower (*p* < 0.05) at LVW compared to LVB (Table 7) as previously reported in 2003 [16]. Male carp from LVW sampled when testicular growth was complete but prior to spawning, showed lower 11-Ketotestosterone (11-Kt) compared to carp from LVB [85]. Because 11-Kt is the sex steroid hormone that controls spermatogenesis and testicular development [86], these data suggest fish in LVW, which were exposed to higher EOC concentrations, and especially those that are estrogenic (Table 6), had relatively impaired gonadal development and thus lower GSI.

Testicular fibrosis, defined as an abnormal thickening of interstitial tissue in the germinal epithelium, has been observed after exposure to environmental stressors [87,88,89], and may be a chronic tissue response to chemical exposure [88], particularly estrogenic compounds [88]. Interstitial thickness of the germinal epithelium was higher in male fish sampled in July (LVW and LVB) compared to the reference site OA sampled in November (Table 7). This was expected after fish released sperm during spawning and testicular lobules contract resulting in thicker intralobular (interstitial) spaces. In the fall, the opposite occurs, because when testes are full of sperm, interstitial spaces are reduced as the lobules expand; however, fish from WB showed a significantly (*p* < 0.05) higher degree of fibrosis (256%) than fish from OA, and even higher values than those from carp collected in July, when the GSI was lower (Table 7). These results indicate an increased deposition of fibrous connective tissue in the testes of WB male carp, confirming that male carp in WB were exposed to environmental stressors affecting reproductive processes [85]. 

### 3.5. Modeling Future EOC Concentrations and Water Temperatures

**Figure 7:**
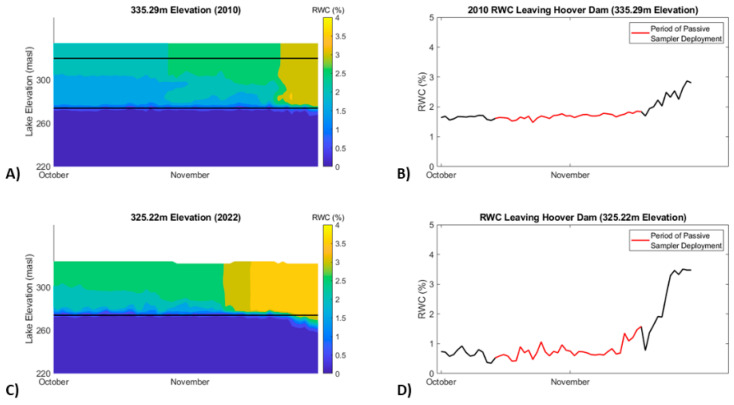
Recycled water concentrations (RWCs), that are the percent of highly treated wastewater effluent from Las Vegas Wash, in October-November. Meters above sea level (masl). (**A**,**C**) are predicted RWCs at the face of Hoover Dam, and (**B**,**D**) are predicted RWCs leaving Hoover Dam. Note lower RWCs in the cooler hypolimnions (blue) and higher RWCs (orange) in the warmer epilimnion in late November (**C**). RWCs leaving Hoover Dam at the lower lake level are lower through mid-November then rising sharply to a maximum of 4% (**D**). Lower lake levels provide less dilution for RWCs resulting in higher RWCs at certain times of the year.

**Figure A1:**
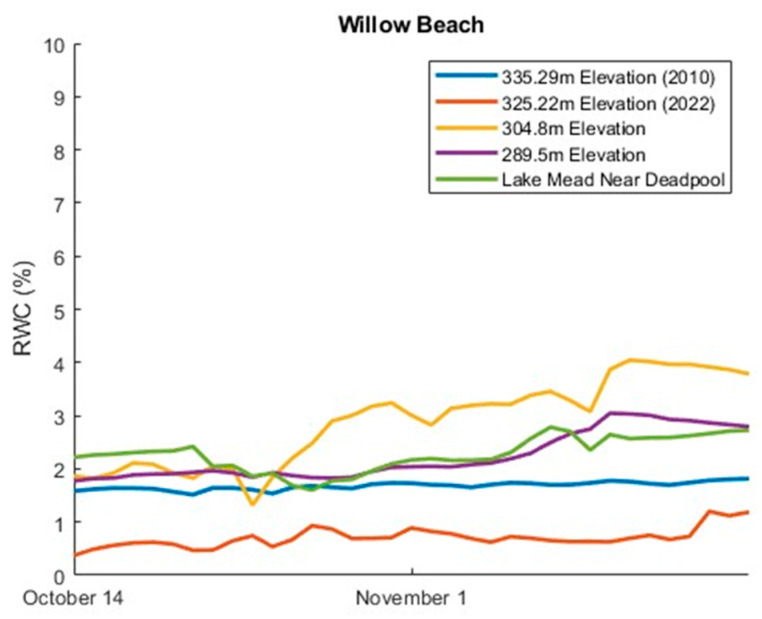
Recycled Water Concentrations (RWC), that are the percent of highly treated wastewater effluent from Las Vegas Wash, in October-November predicted at Willow Beach on the Colorado River below Hoover Dam from a water quality model in 2010 and 2022 and at three different Lake Mead water levels down to deadpool (level where hydro turbines can’t generate electricity). Note the highest RWCs of 4% are predicted in late November at a lower lake level of 304.8 m and the lowest RWCs predicted at a higher lake level of 325.2 m from 2022. This indicates that lower volumes of receiving water in Lake Mead provide less dilution for the wastewater discharged from Las Vegas Wash resulting in higher RWCs.

A water quality model was run to predict recycled water concentration (RWC) at WB below Hoover Dam as Lake Mead levels drop from changes in climate and the prolonged regional drought in the CRB. Since LVW is the primary source of EOCs into Lake Mead and it is 85% treated wastewater [10], knowing how the RWC concentration changes will reflect how EOCs change. To estimate future RWC concentrations, the LM3 water quality model was run using relevant data, including inflow and outflow volumes, water quality measurements, and meteorological parameters from 2010, 2020, and a simulation for a water depth as low as 304.8 m. Results from this LM3 Lake Mead and Lake Mohave water quality model showed mean RWC decreased substantially at WB during the passive sampling period from 1.68% in 2010 to 0.71% in 2022 despite a 10 m drop in Lake Mead (Figure A1). The model predicts values of RWC at WB would increase to 2.89% if Lake Mead dropped to 304.8 m, then decrease slightly to 2.23% at 289.6 m and not change much more, with 2.28% at 278 m being near dead-pool conditions (Figure A1). The highest RWC of 4% is at 304.8 m lake elevation in late November (Figure 7). Overall, the results of the LM3 model suggest that assuming future EOC loading is similar to the current load into Lake Mead, the concentration of any EOC at WB reported from this 2010 study could increase by as much as 135% if the lake water level drops to 304 m. While the maximum increase of EOC concentrations at WB of over 2 times is substantial, this does not raise the concentrations above the available chronic toxicity values for standard EOCs (Table 3) or raise CEC concentrations that have not exceeded screening values to values that do (Table 4). However this does not take into account EOCs or CECs that currently do not have chronic criteria or screening values, new data that may change (e.g., lower) the current criteria or screening values, or the consideration of increased toxicity from mixtures of contaminants. 

While it seems counterintuitive, RWC leaving Hoover Dam was found to be higher at the higher lake elevation corresponding to 2010, versus the lower lake elevation modelled to be representative of conditions in 2022. The seemingly logical conclusion would be that more water available for dilution yields lower RWC; however, Lake Mead is a complicated system and the use of dam outlets that have different elevations drives this result. In 2010, the lake elevation was high enough that both the upper and lower Hoover Dam outlets were utilized, and the model assumes an even split between water volumes released through the two outlets when both outlets are used [46], as both outlets are open when wetted. In May of 2022, the water level of Lake Mead became low enough that the Hoover Dam upper outlet could no longer be utilized, and water was released to Lake Mohave downstream using only the lower outlet in the colder hypolimnion. During October and November, RWC tends to remain near the top of the water column in the warmer epilimnion by the time it travels to the Hoover Dam. The mixed-layer release of 2010 thus had a higher concentration of RWC than the release from 2022, which remained around 1% until the end of November (Figure 7). 

Different seasonal flow patterns, lake stratification, and wind patterns can affect the water column composition of RWC. Deploying the passive samplers during different times of year may yield different results. With lake elevations used in each run of model for 2010, 2022, and the 304.8 m simulation, a strong gradient was exhibited by RWC whereby at 289.6 m and near dead-pool the RWC was mixed more thoroughly in the water column (Figure 7). The depth-averaged RWC was higher at 289.6 m and near dead-pool than it was at 304.8 m; however, due to the location of the intake, the 304.8 m simulation releases water that is high in RWC near the bottom of the gradient. Wind and wave action affect RWC movement in the lake, leading to more thorough mixing in the water column by the time the RWC travels from LVW to Hoover Dam at the lower modelled lake elevations (289.6 m and near dead-pool). Because more vertical mixing occurs in the water column at these lower elevations, the RWC released at 289.6 m and near dead-pool was less than projected at 304.8 m. The mean flow rate between 14 June and 14 July 2010, was 7.64 m^3^/s, and 8.99 m^3^/s in 2022 respectively, indicating a 17.6% increase in mean flow rate. This indicates that additional EOC loading into Lake Mead could have occurred between 2010 and 2022; however, it is difficult to quantify this as treatment technologies at the wastewater treatment plants have also improved between 2010 and 2022.

Warmer releases from Hoover Dam coupled with rising air temperatures directly affect water temperatures at Willow Beach (Table A3). Using a rank sum test, projected end of 21st century climate change causes statistically significant (<0.05) increases in water temperature in all five simulated scenarios of both Lake Mead water level and years compared (Figure 7). Air temperatures rising 4.8 °C, due to climate change, may raise water temperatures at Willow Beach between 0.7–2.1 °C. This warming will become more pronounced relating directly with the drawdown of Lake Mead water levels to 304.8 m. If the water level in Lake Mead falls, releases from Hoover Dam will become epilimnetic, and consequently, warmer and more seasonally variable. There is more mixing in the water column at lake levels of 289.6 m, and near dead pool simulations.

Climate change is raising water temperatures around the world including lakes and streams [90]. Specific effects of warmer water include holding less dissolved oxygen, increasing plankton blooms, altering thermal layering and turnover in lakes, and can increases epizootic fish disease [91]. More general concerns are the alteration of fundamental ecosystem processes and the geographical distribution of species [92]. Warmer water raises metabolism in fish that increases food consumption and respiration and exposure of fish to EOCs in food and water and therefore increases toxicity of some compounds [93]. Dissolved oxygen saturation level concentrations (DOSLC) in Indian streams were predicted to decrease by 2.3% for every 1 °C rise in water temperature so DOSLC could be lowered at WB by over 5% at the end of the Century [94]. However, the ecological response to global warming in aquatic ecosystems is complex and there is uncertainty how systems will change and respond [95].

## 4. Conclusions

A wide range was seen in the number of detections and concentrations of EOCs in water among sites, with LVW showing the most detections at 72, and the highest total sum of EOC concentrations at 1.9 × 10^7^ pg/L. Compared with LVW, total EOC sums were 73% lower in LVB, 93% lower in OA, and 95% lower in WB. LVB is an important area for Razorback Suckers, where this endangered species is exposed to 41 more EOCs at 4Xs higher total concentration than the reference site (OA). The significant EOC concentrations at LVW, along with reduced sperm quality in carp, suggest that over long periods (e.g., decades) of exposures to complex mixtures, well above reference concentrations, are resulting in negative reproductive effects. Although tPCBs in periphyton at WB were an order of magnitude lower than those at the other three sites, and PCBs were not detected in water, higher PCBs in fish could be explained by the much longer exposures in fish at WB. The degree days at WB were 6.7 to 9.7 times lower than at the other three sites, thus lowering the carp growth rate and increasing the lifespan up to 54 years, with a mean age of 44 years, which is ten years older than any site in the CRB. While many EOCs were detected, only four were above screening values. Concentrations of N,N diethyltolumide (DEET) in water exceeded low population relevant screen value (LPRSV) of 1.3 ng/L at all four sites and LVW exceeded the low comprehensive screening value (LCSV) of 23.6 ng/L. Triclosan water concentrations exceeded both the LCSV of 2.5 ng/L and the LPRSV of 2.9 ng/L at WB and LVW. Galaxolide (HHCB) water concentrations at LVW exceeded the LCSV (64.9 ng/L) by 1.5 orders of magnitude and LPRSV (910 ng/L) by 4 times. Individual estrogenic compounds levels were below the lowest levels demonstrated to cause effects in fish (19 ng/L), but total estrogenicity at WB, where a specific source was found in the WBNFH outfall of 3.1 ng/L, was most likely from residual fish food in the fish hatchery discharge.

Effects on biological variables were related to sites with higher environmental contaminants. At LVW, having the highest EOCs of all sites, male carp showed the most abnormal reproductive effects including higher external testicular lesions, fewer reproductively mature sperm, testicular fibrosis, DNA fragmentation and lower GSIs, sperm motility, and viability. EOCs in LVW discharged into LVB can affect other fish downstream in Boulder Basin and the Colorado River below Hoover Dam, including WB, if regional drought continues and water levels in Lake Mead lower from drier climates. Although EOCs were generally similar between OA and WB, fish from WB have been exposed much longer because of lower growth and longer lifespans (up to 54 years) resulting in higher PCB concentrations and more effects on biological variables than at OA, including lower liver glycogen, external testicular lesions, and an order of magnitude higher testicular PgCAs. 

Water levels in Lake Mead have dropped 31 m, from 2010 to current levels in 2023 because of prolonged regional drought (>two decades) in the Colorado River Basin. This drop decreases the amount of water available to dilute the LVW inflow into Lake Mead and downstream through Hoover Dam into the Colorado River and into Lake Mohave. Water quality modeling predicts that a proportion of LVW (RWC) concentrations at the SNWA drinking water intake in the receiving waters of Boulder Basin have increased by 72% from 1.68% to 2.89%. Further drought influenced by continued global warming could increase RCW concentration to as much as 3.6%, which could increase EOC concentrations in Boulder Basin where Razorback Suckers are exposed. At WB, EOC concentrations could increase up to 135%, which could affect fish health and reproduction. Air temperatures rising 4.8 °C at the end of the 21st century may raise water temperatures at Willow Beach between 0.7–2.1 °C, which could increase growth rates in carp. This rise in metabolism can also increase food consumption and respiration that in turn escalate EOC exposure and accumulation particularly for slowly metabolized EOCs like PCBs. 

## Figures and Tables

**Table 1:** Total polychlorinated biphenyls ^1^ detected from various media in 2008 from matrices sampled from the Colorado River and sites in Willow Beach National Fish Hatchery, Hatchery, Arizona, USA.

Site ^2^	Water ^3^	Sediment	Periphyton	Trout	Razorback	Raceway	Raceway
				Feed	Feed	Rubber	Gap Filler ^4^
1	ND ^5^	1.03	1.62	NA ^5^	NA	NA	NA
2	ND	1.22	0.05	NA	NA	NA	NA
3	ND	ND	4.8	3.4	9.1	81.9	32.1
4	ND	ND	ND	NA	NA	NA	NA
5	ND	ND	5.92	NA	NA	NA	NA

^1^ ng/g dry weight. ^2^ See Figure 1. ^3^ Method detection limit, 460 pg/L. ^4^ Detected from one of three raceways sampled. ^5^ Not detected (ND) and not applicable (NA).

**Table 2:** Comparison of Environmental Organic Compounds (EOC) in water from sites on the Colorado River below Hoover Dam and Lake Mead, 2010.

		Overton Arm		Willow Beach ^1^			Las Vegas Bay		Las Vegas Wash	
EOC Group ^2^	EOCs ^3^	Detections ^3^	Sum ^4^	Detections	Sum	±SD	Detections	Sum	Detections	Sum
OC	32	15	581	21	583	80	23	3732 ^5^	24	9247
PCB	1	ND ^6^	0	ND	0	0	1	525	1	420
PBDE	5	3	27	5	106	49	4	282	5	1385
PAH	34	1	25	12	1223	424	15	2,053	17	2532
Waste Water	44	5	1,291,000	9	987,000	225	15	5,088,000	16	18,672,000
Antimicrobials	2	ND	0	2	3	1.8	2	5	2	24
Fragrances	3	ND	0	ND	0	_	2	94	3	3,910
Pharmaceuticals	9	ND	0	ND	0	_	3	1,720	4	12,240
Total EOCs	130	24	1,291,633	49	988,915	225	65	5,096,411	72	18,701,750

^1^ Mean of 5 stations (see inset of Figure 1). ^2^ Organochlorines (OC); Polychlorinated biphenyls (PCB); Polybrominated diphenyl ethers (PBDE); Polyaromatic hydrocarbons (PAH). ^3^ Indicates the number of different EOCs detected and the number detected. ^4^ Sum total of concentrations in pg/L. ^5^ Mean of surface and bottom concentrations. ^6^ ND = not detected.

**Table 6:** Total estrogen receptor agonist activity (ERAA) of environmental organic compound (EOC) concentrations (pg/L) detected in 2010 in water from sites in the Lake Mead National Recreation Area, Nevada and Arizona, United States; Comparison is made to 17 α-Ethinylestradiol (αERAA).

		Overton Arm		Willow Beach ^1^		Las Vegas Bay ^2^		Las Vegas Wash	
EOC	αERAA ^3^	EOC	ERAA	EOC	ERAA	EOC	ERAA	EOC	ERAA
Lindane	0.00306	ND ^4^	0	ND	0	40	0.1224	140	0.4284
Chlorpyrifos	0.0125	110	1.375	ND	0	39.5	0.4938	130	1.625
Cis chlordane	0.0191	ND	0	2	0.0458	46.5	0.8882	58	1.1078
Dieldrin	0.0181	6.3	0.114	17	0.308	60.5	1.0951	85	1.5385
Benz[a]anthracene	0.0372	ND	0	ND	0	ND	0	37	1.3764
Fluoranthene	0.00745	ND	0	250	1.863	185	1.3783	500	3.725
Benzo-b-fluoranthene	0.012	ND	0	ND	0	ND	0	47	0.564
Heptachlor epoxide	0.00739	6.9	0.051	15	1.796	41.5	0.3067	ND	0
*o,p*′-DDD	0.236	ND	0	21	4.956	85	20.06	78	18.408
*P,P*′-DDD	0.0728	5.4	0.393	10	0.728	105	7.644	ND	0
*p,p*′-DDE	0.0677	11	0.745	10	0.677	185	12.5245	58	3.9266
*o,p*′-DDT	0.385	ND	0	ND	0	7.6	2.926	140	53.9
*p,p*′-DDT	0.19	ND	0	7.7	1.463	ND	0	120	22.8
Endosulfan	0.0295	ND	0	ND	0	75	2.2125	1800	53.1
Benzonophenone	0.04	ND	0	ND	0	7000	280	130,000	5200
Total			2.7		11.8		329.7		5362.5

^1^ Station WB3 (see inset Figure 1). ^2^ Mean from surface and bottom concentrations. ^3^ 17α-Ethinylestradiol is the comparison compound [62]. ^4^ Not detected; below the method detection limit.

**Table 7:** Biological condition of common carp collected from Lake Mead in 2010. Values shown are mean ± SEM; n = sample size. *K*, Fulton condition factor; Hct, Hematocrit; GSI, gonadosomatic index; PgCA, pigmented cell aggregates. Statistical differences at *p* ≥ 0.05 between LVB and LVW, and OA and WB denoted by different letters.

Biological Traits	July		November	
	LVB	LVW	OA	WB
n	30	29	23	17
External lesions;% of fish	20	3.5	13	0
Length (mm)	528.1 ± 7.9 ^a^	431.0 ± 6.0 ^b^	496.5 ± 11.4 ^a^	554.2 ± 10.0 ^b^
Weight (g)	2021.5 ± 109.2 ^a^	1045 ± 43.5 ^b^	1630.9 ± 154.9 ^a^	2787.4 ± 182.3 ^b^
*K*	1.3 ± 0.01	1.3 ± 0.02	1.26 ± 0.02 ^a^	1.6 ± 0.1 ^b^
Hct (%)	39.5 ± 1.1	41.4 ± 0.9	39.1 ± 0.9	42.2 ± 2.2
GSI (%)	3.9 ± 0.2 ^a^	2.5 ± 0.2 ^b^	7.4 ± 0.3	6.9 ± 0.5
**Testes**				
External lesions (%)	3.3	6.9	8.7	11.8
PgCA area (%)	0.9 ± 0.3 ^a^	0.6 ± 0.3 ^b^	0.13 ± 0.1 ^a^	1.5 ± 0.5 ^b^
Interstitium thickness (µm)	41.7 ± 2.7	46.5 ± 3.0	21.8 ± 1.9 ^a^	56.0 ± 6.0 ^b^
Focal Granuloma (% of fish)	36.7 ^a^	0 ^b^	26.1	35.3
**Liver**				
PgCA area (%)	1.6 ± 0.4	0.4 ± 0.3	0.8 ± 0.2	0.5 ± 0.2
Lipid deposition (score)	1.5 ± 0.1	1.4 ± 0.1	1.3 ± 0.1 ^a^	1.8 ± 0.2 ^b^
Glycogen deposition (score)	2.2 ± 0.1	2.2 ± 0.1	2.4 ± 0.1 ^a^	1.8 ± 0.3 ^b^
**n**	**30**	**29**	**23**	**16**
**Sperm Quality**				
Abnormal sperm (%)	4.2 ± 0.4	4.3 ± 0.3	3.8 ± 0.4	4.0 ± 0.4
Progressive motility (%)	59.3 ± 2 ^a^	40.1 ± 4.7 ^b^	78.1 ± 3.2	74.7 ± 3.4
Total motility (%)	60.7 ± 2.9 ^a^	41.9 ± 4.7 ^b^	79.4 ± 3.3	76.9 ± 3.3
Mitochondrial function (%)	71.8.0 ± 4.7	88.41.2	97.2 ± 0.3	96.1 ± 1.1
Counts (#)	1.5 × 10^10^ ± 1.0 × 10^9^	1.1 × 10^10^ ± 1.6 × 10^9^	2.9 × 10^10^ ± 3.8 × 10^9^	2.5 × 10^10^ ± 2.3 × 10^9^
DNA fragmentation CV (%)	2.0 ± 0.1 ^a^	4.3 ± 0.3 ^b^	2.5 ± 0.2 ^a^	1.9 ± 0.1 ^b^
Viable (%)	91.2 ± 1.2 ^a^	88.7 ± 1.0 ^b^	96.9 ± 0.2	96.7 ± 0.4
Total apoptotic (%)	15.1 ± 2.0	17.9 ± 2.0	4.95 ± 0.4	5.7 ± 0.7
Over haploid (%)	4.1 ± 0.4 ^a^	8.8 ± 1.8 ^b^	1.6 ± 0.1	1.5 ± 0.1
* ATP	0.021 ± 0.002 ^a^	0.015 ± 0.003 ^b^	0.017 ± 0.002 ^a^	0.033 ± 0.005 ^b^

* Calculated with half of the samples from LVW due to a sample shipping issue.

## Data Availability

Data are publicly available: [26,35,36].

## Appendix A

**Table A1:** List of individually targeted contaminants within each analytical group analyzed by either semipermeable membrane device samplers (S) or Polar Organic Chemical Integrative Samplers (P).

Organochlorine Pesticides (S)	Polycyclic Aromatic Hydrocarbons (S)	Waste Water Chemicals (S, P)	Pharmaceuticals (P)
Trifluralin	Naphthalene	Isopropylbenzene (cumene) (S)	Azithromycin
Hexachlorobenzene (HCB)	Acenaphthylene	1,4-Dichlorobenzene (S)	Clarithromycin
Pentachloroanisole (PCA)	Acenaphthene	d-Limonene (S)	Clindamycin
Tefluthrin	Fluorene	Acetophenone (S)	Roxithromycin
alpha-Benzenehexachloride (a-BHC)	Phenanthrene	Isophorone (P)	Hydrocodone
Lindane	Anthracene	Triethyl phosphate (P)	Pseudoephedrine
beta-Benzenehexachloride (b-BHC)	Fluoranthene	Camphor (P)	Urobilin
Heptachlor	Pyrene	Menthol (P)	MDMA (Ecstasy)
delta-Benzenehexachloride (d-BHC)	Benz[a]anthracene	Methyl salicylate (P)	Methamphetamine
Dacthal	Chrysene	Dichlorvos (P)	
Chlorpyrifos	Benzo[b]fluoranthene	Isoquinoline (P)	**Yeast Estrogen Screen (P)**
Oxychlordane	Benzo[k]fluoranthene	Indole (P)	Total Estrogenicity
Heptachlor Epoxide	Benzo[a]pyrene	N,N-diethyltoluamide (P)	
trans-Chlordane	Indeno[i,2,3-c,d]pyrene	Diethyl phthalate (P)	
trans-Nonachlor	Dibenz[a,h]anthracene	p-tert-Octylphenol (P)	
o,p’-DDE	Benzo[g,h,i]perylene	Benzophenone (P)	
cis-Chlordane	Benzo[b]thiophene	Tributyl phosphate (P)	
Endosulfan	2-methylnaphthalene	Ethyl citrate (P)	
p,p’-DDE	1-methylnaphthalene	Cotinine (P)	
Dieldrin	Biphenyl	Celestolide (P)	
o,p’-DDD	1-ethylnaphthalene	Prometon (P)	
Endrin	1,2-dimethylnaphthalene	Phantolide (P)	
cis-Nonachlor	4-methylbiphenyl	4-Octylphenol (P)	
o,p’-DDT	2,3,5-trimethylnaphthalene	Tri(2-chloroethyl) phosphate (P)	
p,p’-DDD	1-methylfluorene	N-butyl benzenesulfonamide (P)	
Endosulfan-II	Dibenzothiophene	Tris-(2-chloropropyl)phosphate (P)	
p,p’-DDT	2-methylphenanthrene	Diazinon (P)	
Endosulfan Sulfate	9-methylanthracene	Tris-(1,3-dichloro-2-propyl)phosphate (P)	
p,p’-Methoxychlor	3,6-dimethylphenanthrene	Musk Ambrette (P)	
Mirex	2-methylfluoranthene	Carbazole (P)	
cis-Permethrin	Benzo[b]naphtho [2,1-d]thiophene	Caffeine (P)	
trans-Permethrin	Benzo[e]pyrene	Traseolide (P)	
	Perylene	Galaxolide (P)	
**Polychlorinated Biphenyls (S)**	3-methylcholanthrene	Tonalide (P)	
Total PCBs		Musk Xylene (P)	
		Carbaryl (P)	
**Polybrominated dipenyl ethers (S)**		Metalaxyl (P)	
PBDE-28		Bromacil (P)	
PBDE-47		Anthraquinone (P)	
PBDE-99		Musk Ketone (P)	
PBDE-100		Chlorpyrifos (P)	
PBDE-153		Triclosan (S)	
		Methyl Triclosan (S)	
		Tri(dichloroisopropyl) phosphate (P)	
		Tri(butoxyethyl) phosphate (P)	
		Triphenyl phosphate (P)	
		Tris-(2-ethylhexyl)phosphate (P)	
		Diethylhexylphthalate (P)	
		Cholesterol (P)	

**Table A2:** Risk quotient (RQ) and PNEC1 estimates for aquatic life to pharmaceuticals (ng/L) detected in 2010 from Lake Mead National Recreation Area, U.S.

Pharmaceutical	Las Vegas Bay Surface Water	Las Vegas Bay Bottom Sediment	Las Vegas Wash Water	PNEC ^1^	RQ Value ^2^	Surface Water^max 3^
Azrithromycin	ND ^4^	ND	4.4	820,000	>0.01 ^5^	2356
Clindamycin	1.3	0.45	2.5	230,000	>0.01	11
Hydrocodone	0.57	0.53	4.7	2,500,000	>0.01	10
Methamphetamine	0.26	0.17	0.64	1,100,000	>0.01	63

^1^ Predicted no effect concentration is the concentration of a chemical below which no adverse effects of exposure in an ecosystem are measured. ^2^ Risk quotients are metrics used in environmental risk assessments, being calculated by comparing the measured environmental concentration to the PNEC. RQ’s below 1.0 pose little risk to aquatic biota. ^3^ Maximum concentration detected in surface water. ^4^ Not detected or below the method detection limit. ^5^ Risk quotient value at >0.01 is low risk.

**Table A3:** Projected water temperatures at Willow Beach during October and November under various lake levels comparing existing and estimated late 21st century air temperatures.

Lake Mead Water Level (M)	Climate Scenario	Median Water Temperature (°C)	*p*-Value	Statistically Significant Difference?
335.3	Historic	19.4 ±1.2	10^−4^	yes
	RCP 8.5/late century ^1^	20.1 ± 1.1		
325.2	Historic	15.5 ± 0.34	10^−10^	yes
	RCP 8.5/late century	16.3 ± 0.43		
304.8	Historic	20.1 ± 1.1	10^−6^	yes
	RCP 8.5/late century	21.2 ± 1.2		
289.6	Historic	19.1 ± 1.9	10^−5^	yes
	RCP 8.5/late century	21.5 ± 1.8		
Near Deadpool 278.0	Historic	18.3 ± 1.9	10^−5^	yes
	RCP 8.5/late century	20.4 ± 2.0		

^1^ Climate projections for 2070-2099 from the Climate Model Intercomparison Project (accessed 26 September 2024: https://www.wcrp-climate.org/wgcm-cmip).

## References

[1] Rosen M.R., Turner K., Goodbred S.L., Miller J.M. (2012). A Synthesis of Aquatic Science for Management of Lakes Mead and Mohave.

[2] Macrotrends Las Vegas Metro Area Population 1950–2023. http://www.macrotrends.net/cities/23043/Las/Vegas/population.

[3] Holdren G.C., Turner K. (2010). Characteristics of Lake Mead, Arizona-Nevada. Lake Reserv. Manag..

[4] Patiño R., VanLandeghem M.M., Goodbred S.L., Orsak E., Jenkins J.A., Echols K.R., Rosen M.R., Torres L. (2015). Novel associations between contaminant body burdens and biomarkers of reproductive condition in male Common Carp along multiple gradients of contaminant exposure in Lake Mead National Recreation Area, USA. Gen. Comp. Endocrinol..

[5] Wong W.H., Gerstenberger S.L., Baldwin W., Moore B. (2012). Settlement and growth of quagga mussels (*Dreissena rostriformis bugensis* Andrusov, 1897) in Lake Mead, Nevada-Arizona, USA. Aquat. Invasions.

[6] Allen T. The Quagga Conundrum. University of Nevada, Las Vegas 2009. https://www.unlv.edu/news/article/quagga-conundrum.

[7] Goodbred S., Rosen M.R., Patiño R., Alvarez D., Echols K., King K., Umek J. (2021). Movement of synthetic organic compounds in the food web after the introduction of invasive quagga mussels (*Dreissena bugensis*) in Lake Mead, Nevada and Arizona, USA. Sci. Total Environ..

[8] Tietjen T. Drought and Water Quality in Lake Mead. North American Lake Management Society, Lakeline 2015. https://www.nalms.org/wp-content/uploads/LakeLine/35-4/Articles/35-4-11.pdf.

[9] Jenkins J.A., Eilts B.E., Guitreau A.M., Figiel C.R., Draugelis-Dale R.O., Tiersch T.R. (2011). Sperm quality assessments for endangered razorback suckers *Xyrauchen texanus*. Reproduction.

[10] Benotti M.J., Stanford B.D., Snyder S.A. (2010). Impact of drought on wastewater contaminants in an urban water supply. J. Environ. Qual..

[11] Nasa Earth Observatory Lake Mead Keeps Dropping. https://earthobservatory.nasa.gov/images/150111/lake-mead-keeps-dropping.

[12] Williams A.P., Cook E.R., Smerdon J.E., Cook B.I., Abatzoglou J.T., Bolles K., Baek S.H., Badger A.M., Vivneh B. (2020). Large contribution from anthropogenic warming to an emerging North American megadrought. Science.

[13] Yau F., Livneh B., Rajagopalan B., Wang J., Cretaux J., Wada Y., Berge-Nguyen M. (2023). Satellites reveal widespread decline in global lake water storage. Science.

[14] Hannoun D., Tietjen T. (2023). Lake management under severe drought: Lake Mead, Nevada, Arizona. J. Am. Water Resour. Assoc..

[15] Bevans H.E., Goodbred S.L., Miesner J.F., Watkins S.A., Gross T.S., Denslow N.D., Schoeb T. (1996). Synthetic Organic Compounds and Carp Endocrinology and Histology in Las Vegas Wash and Las Vegas and Callville Bays of Lake Mead, Nevada 1992 and 1995.

[16] Patiño R., Goodbred S.L., Draugelis-Dale R., Barry C.E., Foott J.S., Wainscott M.R., Gross T.S., Covay K.J. (2003). Morphometric and histopathological parameters of gonadal development in adult common carp from contaminated and reference sites in Lake Mead, Nevada. J. Aquat. Anim. Health.

[17] Goodbred S.L., Smith S.B., Greene P.S., Rauschenberger R.H., Bartish T.H. Reproductive and Endocrine Biomarkers in Largemouth Bass (Micropterus salmoides) and Common Carp (Cyprinus carpio) from United States Waters.

[18] Jenkins J.A., Rosen M.R., Draugelis-Dale R.O., Echols K.R., Torres L., Wieser C.M., Kersten C.A., Goodbred S.L. (2018). Sperm quality biomarkers complement reproductive and endocrine parameters in investigating environmental contaminants in common carp (*Cyprinus carpio*) from the Lake Mead National Recreation Area. Environ. Res..

[19] Goodbred S.L., Patiño R., Torres L., Echols K.R., Jenkins J.A., Rosen M.R., Orsak E. (2015). Are endocrine and reproductive biomarkers altered in contaminant-exposed wild male Largemouth Bass (*Micropterus salmoides*) of Lake Mead, Nevada/Arizona, USA?. Gen. Comp. Endocrinol..

[20] Hinck J.E., Blazer V.B., Denslow N.D., Echols K.R., Gross T.S., May T.W., Anderson P.J., Coyle J.J., Tillitt D.E. (2007). Chemical contaminants, health indicators, and reproductive biomarker responses in fish from the Colorado River and its tributaries. Sci. Total Environ..

[21] Mueller G., Marsh P.C., Knowles G., Wolters T. (2000). Distribution, movements, and habitat use of razorback sucker (*Xyrauchen texanus*) in a lower Colorado River reservoir, Arizona-Nevada. West. N. Am. Nat..

[22] Albrecht B., Mohn H.E., Kegerries R., McKinstry M.C., Rogers R., Francis T., Hines B., Stolberg J., Ryden D., Elverud D. (2018). Use of inflow areas in two Colorado River Basin reservoirs by the endangered Razorback Sucker (*Xyrauchen texanus*). West. N. Am. Nat..

[23] Alvarez D.A., Cranor W.L., Perkins S.D., Clark R.C., Smith S.B. (2008). Chemical and toxicological assessment of organic contaminants in surface water using passive samplers. J. Environ. Qual..

[24] Alvarez D.A., Rosen M.R., Perkins S.D., Cranor W.L., Schroeder V.L., Jones-Lepp T.L. (2012). Bottom sediment as a source of organic contaminants in Lake Mead, Nevada, USA. Chemosphere.

[25] Rosen M.R., Alvarez D.A., Goodbred S.L., Leiker T.J., Patiño R. (2010). Sources and distribution of organic compounds using passive samplers in Lake Mead National Recreation Area, Nevada and Arizona, and their implications for potential effects on aquatic biota. J. Environ. Qual..

[26] Alvarez D.A., Echols K.R. (2024). Contaminants measured in multiple environmental media in the Lake Mead National Recreation Area NV/AZ, USA in 2010. U.S. Geol. Surv. Data Release.

[27] Hannoun D., Tietjen T., Brooks K. (2021). The potential effects of climate change and drawdown on a newly constructed drinking water intake: Study case in Las Vegas, NV, USA. Water Util. J..

[28] Hannoun D., Belding J., Tietjen T., Devaney R. (2022). Assessing treatability with simulated lake drawdown: Quantifying drought-driven turbidity in source water. AWWA Water Sci..

[29] Alvarez D.A. (2010). Guidelines for the Use of the Semipermeable Membrane Device (SPMD) and the Polar Organic Chemical Integrative Sampler (POCIS) in Environmental Monitoring Studies.

[30] Huckins J.N., Petty J.D., Booij K. (2006). Monitors of Organic Chemicals in the Environment: Semipermeable Membrane Devices.

[31] Valentine J.J. (1983). Colorado River Endangered Fish Hatchery Feasibility Study.

[32] Peterman P.H., Orazio C.E., Echols K.R. (2006). Basic alumina flash chromatographic separation of bulk ortho-PCBs from on-ortho-PCBs, PBDEs, PCDFs, PCDDs, PCDTs, OCPs, and PCTs. Organohalogen Compd..

[33] Uses of Fishes in Research Committee (Joint Committee of the American Fisheries Society, the American Institute of Fishery Research Biologists, and the American Society of Ichthyologists and Herpetologists) (2014). Guidelines for the Use of Fishes in Research.

[34] Luna L.G. (1992). Histopathological Methods and Color Atlas of Special Stains and Tissue Artifacts.

[35] (2023). Sperm quality biomarkers from common carp (*Cyprinus carpio*) in spring and fall 2010 from Lake Mead, Arizona and Nevada, USA. U.S. Geol. Surv. Data Release.

[36] Castille A.E., Jenkins J.A. (2023). Measures of adenosine triphosphate in sperm from common carp (*Cyprinus carpio*) in spring and fall 2010 from Lake Mead, Arizona and Nevada, USA. U.S. Geol. Surv. Data Release.

[37] Jenkins J.A., Olivier H.M., Draugelis-Dale R.O., Eilts B.E., Torres L., Patiño R., Nilsen E., Goodbred S.L. (2014). Assessing reproductive and endocrine parameters in male largescale suckers (*Catostomus macrocheilus*) along a contaminant gradient in the lower Columbia River, USA. Sci. Total Environ..

[38] Crissman H.A., Steinkamp J.A. (1973). Rapid simultaneous measurement of DNA, protein and cell volume in single cells from large mammalian cell populations. J. Cell Biol..

[39] Jenkins J.A., Tiersch T.R., Green C.C. (2011). Male germplasm in relation to environmental conditions: Synoptic focus on DNA. Cryopreservation in Aquatic Species.

[40] Ringner M. (2008). What is principal component analysis?. Nat. Biotechnol..

[41] SAS Institute, Inc (2018). SAS/STAT15.1 User’s Guide.

[42] Horn J.L. (1965). A rationale and test for the number of factors in factor analysis. Psychometrica.

[43] Franklin S.B., Gibson D.J., Robertson P.A., Pohlmann J.T., Fralish J.S. (1995). Parallel analysis: A method for determining significant principal components. J. Veg. Sci..

[44] Hodges B., Dallimore C. (2016). Aquatic Ecosystem Model: AEM3D, v1.0..

[45] Hannoun D., Tietjen T., Brooks K. (2022). The influence and implications of climate change on water quality in a large water reservoir in the southwest, USA. Am. J. Clim. Change.

[46] Preston A., Hannoun I.A., List E.J., Rackley I., Tietjen T. (2014). Three-dimensional management model for Lake Mead, Nevada, Part 2: Findings and applications. Lake Reserv. Manag..

[47] Kalansky J., Sheffield A., Cayan D., Pierce D. Climate conditions in Clark County, NV: An Evaluation of Historic and Projected Future Climate Using Global Climate Models; 159p. https://www.wucaonline.org/assets/pdf/pubs-clark-county-climate-report.pdf.

[48] Li X., Dong S., Wang P., Su X., Fu J. (2019). Polychlorinated biphenyls are still alarming persistent organic pollutants in marine-origin animal feed (fishmeal). Chemosphere.

[49] Gallego E., Grimalt J.O., Bartrons B., Lopez J.F., Camerero L., Catalan J., Stuchlik E., Battarbee R. (2007). Altitudinal gradients of PBDEs and PCBs in fish from European high mountain lakes. Environ. Sci. Technol..

[50] Vives I., Grimalt J.O., Ventura M., Catalan J., Rosseland B.O. (2005). Age dependence of the accumulation of organochlorine pollutants in brown trout (*Salmo trutta*) from a remote high mountain lake (Redo, Pyrenees). Environ. Pollut..

[51] Vives I., Grimalt J.O., Catalan J., Rosseland B.O., Battarbee R.W. (2004). Influence of altitude and age in the accumulation of organochlorine compounds in fish from high mountain lakes. Environ. Sci. Technol..

[52] Weber M.J., Brown M.L., Wahl D.H., Shoup D.E. (2015). Metabolic theory explains latitudinal variation in common carp populations and predicts responses to climate change. Ecosphere.

[53] Froese R., Pauly D., FishBase World Wide Web Electronic Publication, Version (02/2024). www.fishbase.org.

[54] Baldigo B.P., Sloan R.J., Smith S.B., Denslow N.D., Blazer V.B., Gross T.S. (2006). Polychlorinated biphenyls, mercury, and potential endocrine disruption in fish from the Hudson River, New York, USA. Aquat. Sci..

[55] Richman L.A., Somers K. (2010). Monitoring metal and persistent organic contaminant trends through time using quagga mussels (*Dreissena bugensis*) collected from the Niagara River. J. Great Lakes Res..

[56] Gefell D.J., Banda J.A., Moore J.N., Secord A.L., Tucker W.A. (2019). Ecological Hazard Assessment of Contaminants of Emerging Concern in the U.S. Great Lakes Basin: Part B-Supplemental Document: Technical Resources for Ecological Hazard Assessments of Contaminants of Emerging Concern in Freshwater Fish.

[57] Fan B., Wang X., Li J., Gao X., Li W., Huang Y., Liu Z. (2019). Deriving aquatic life criteria for galaxolide (HHCB) and ecological risk assessment. Sci. Total Environ..

[58] USEPA National Recommended Water Quality Criteria. https://www.epa.gov/wqc/national-recommended-water-quality-criteria-aquatic-life-criteria-table.

[59] Deo R.P. (2014). Pharmaceuticals in the surface water of the USA: A Review. Curr. Environ. Health Rep..

[60] Mathon B., Ferreol M., Coquery M., Choubert J., Chovenlon J., Miege C. (2021). Direct photodegradation of 36 organic micropollutants under simulated solar radiation: Comparison with free-water surface constructed wetland and influence of chemical structure. J. Hazard. Mater..

[61] Delistraty D. (1997). Toxic equivalency factor approach for risk assessment of polycyclic aromatic hydrocarbons. Toxicol. Environ. Chem..

[62] USEPA Endocrine Disruptor Screening Program Estrogen Receptor Bioactivity. https://www.epa.gov/endocrine-disruption/endocrine-disruptor-screening-program-edsp-estrogen-receptor-bioactivity.

[63] Matsumoto T., Kobayashi M., Moriwaki T., Kawai S., Watabe S. (2004). Survey of estrogenic activity in fish feed by yeast estrogen-screen assay. Comp. Biochem. Physiol. Part C Toxicol. Pharmacol..

[64] Jarošová B., Bláha L., Giesy J.P., Hilscherová K. (2014). What level of estrogenic activity determined by in vitro assays in municipal waste waters can be considered as safe?. Environ. Int..

[65] Snyder S.A., Keith L.H., Verbrugge D.A., Snyder E.M., Gross T.S., Kannan K., Giesy J.P. (1999). Analytical methods for detection of selected estrogenic compounds in aqueous mixtures. Environ. Sci. Technol..

[66] Krein A., Pailler J.-Y., Guignard C., Gutleb A.C., Hoffmann L., Meyer B., Keßler S., Berckmans P., Witters H.E. (2012). Determination of estrogen activity in river waters and wastewater in Luxembourg by chemical analysis and the yeast estrogen screen assay. Environ. Pollut..

[67] Lavado R., Loyo-Rosales J.E., Floyd E., Kolodziej E.P., Snyder S.A., Sedlak D.L., Schlenk D. (2009). Site-specific profiles of estrogenic activity in agricultural areas of California’s inland waters. Environ. Sci. Technol..

[68] Marr C.L.H. (2007). Endocrine Disruption in Razorback Sucker and Common Carp on National Wildlife Refuges along the Lower Colorado River.

[69] Jenkins J.A., Rosen M.R., Draugelis-Dale R.O., Echols K.R., Torres L., Wieser C.M., Kersten C.A., Goodbred S.L. (2018). Sperm quality, reproductive, endocrine parameters and environmental contaminants in common carp from Lake Mead National Recreation Area (1999–2006). U.S. Geol. Surv. Data Release.

[70] Hatef A., Alavi S.M.H., Abdulfatah A., Fontaine P., Rodina M., Linhart O. (2012). Adverse effect of bisphenol A on reproductive physiology in male goldfish at environmentally relevant concentrations. Ecotoxicol. Environ. Saf..

[71] Thorpe K.L., Cummings R.I., Hutchinson T.H., Scholze M., Brighty G., Sumpter J.P., Tyler C.R. (2003). Relative potencies and combination effects of steroidal estrogens in fish. Environ. Sci. Technol..

[72] Golshan M., Hatef A., Socha M., Milla S., Butts I.A.E., Carnevali O., Rodina M., Sokolowska-Mikolajczyk M., Fontaine P., Linhart O. (2015). Di-(2-ethylhexyl)-phthalate disrupts pituitary and testicular hormonal functions to reduce sperm quality in mature goldfish. Aquat. Toxicol..

[73] Schlenk D., Schlenk D., Benson W.H. (2001). General Mechanisms of Toxicity. Target Organ Toxicity in Marine and Freshwater Teleosts.

[74] Wolf J.C., Wolfe M.J. (2005). A brief overview of nonneoplastic hepatic toxicity in fish. Toxicol. Pathol..

[75] De Boeck G., Vlaeminck A., Van der Linden A., Blust R. (2000). Salt stress and resistance to hypoxic challenges in the common carp (*Cyprinus carpio*). J. Fish Biol..

[76] Ortiz-Delgado J.B., Segner H., Arellano J.M., Sarsquete C. (2007). Histopathological alterations, EROD activity, CYP1A protein and biliary metabolites in gilthead seabream *Sparus aurata* exposed to benso(a)pyrene. Histol. Histopathol..

[77] Jenkins J.A., Draugelis-Dale R.O. (2006). Bioindicators from Mosquitofish (Gambusia affinis) Sampled from the Imperial Valley in Southern California.

[78] Fournie J.W., Wolfe M.J., Wolf J.C., Courtney L.A., Johnson R.D., Hawkins W.E. (2005). Diagnostic criteria for proliferative thyroid lesions in bony fishes. Toxicol. Pathol..

[79] Micale V., Perdichizzi F. (1990). Gonadal responsiveness to photoperiod extension in captivity-born *Sparus aurata* (L.) during the male phase. Boll. Di Zool..

[80] Wolke R.E. (1992). Piscine macrophage aggregates: A review. Annu. Rev. Fish Dis..

[81] Lister A., Van der Kraak G. (2002). Modulation of goldfish testicular testosterone production in vitro by tumor necrosis factor alpha, interleukin-1beta, and macrophage conditioned media. J. Exp. Biol..

[82] Loir M., Sourdaine P., Mendis-Handagama S.M., Jegou B. (1995). Cell-cell interactions in the testis of teleosts and elasmobranchs. Microsc. Res. Tech..

[83] Hacker-Klom U.B., Meistrich M.L., Gohde W. (1986). Effect of doxorubin and 4′-epi-doxorubicin on mouse spermatogenesis. Mutat. Res. Fundam. Mol. Mech. Mutagen..

[84] Jenner N.J., Ostrander G.K., Kavanagh T.J., Livesey J.C., Shen M.W., Kim S.C., Holmes E.H. (1990). A flow cytometric comparison of DNA content and glutathione levels in hepatocytes of english sole (*Parophyrs vetulus*) from areas of differing water quality. Arch. Environ. Contam. Toxicol..

[85] Patiño R., Rosen M.R., Orsak E.L., Goodbred S.L., May T.W., Alvarez D., Echols K.R., Wieser C.M., Ruessler S., Torres L. (2012). Patterns of metal composition and biological condition and their association in male common carp across an environmental contaminant gradient in Lake Mead National Recreation Area, Nevada and Arizona, USA. Sci. Total Environ..

[86] Borg B. (1994). Androgens in teleost fishes. Comp. Biochem. Physiol. Part C Pharmacol. Toxicol. Endocrinol..

[87] Barse A.V., Chakrabarti T., Ghosh T.K., Pal A.K., Kumar N., Raman R.P., Jadhao S.B. (2010). Vitellogenin induction and histo-metabolic changes following exposure of *Cyprinus carpio* to methyl paraben. Asian-Australas. J. Anim. Sci..

[88] Kaptaner B., Unal G. (2011). Effects of 17a-ethynylestradiol and nonylphenol on liver and gonadal apoptosis and histopathology in *Chalcalburnus Tarichi*. Environ. Toxicol..

[89] Vazquez G.R., Meijide F.J., Da Cuna R.H., Lo Nostro F.L., Piazza Y.G., Babay P.A., Trudeau V.L., Maggese M.C., Guerrero G.A. (2009). Exposure to waterborne 4-tert-octylphenol induces vitellogenin synthesis and disrupts testis morphology in the South American freshwater fish *Cichlasoma dimerus* (Teleostei, Perciformes). Comp. Biochem. Physiol. Part C Toxicol. Pharmacol..

[90] Havens K., Jeppesen E. (2018). Ecological responses of lakes to climate change. Water.

[91] Ruttner F. (2020). Fundamentals of Limnology.

[92] Poff N.L., Brinson M.M., Day J.W. (2002). Aquatic ecosystems and global climate change. Pew Cent. Glob. Clim. Change Arlingt. VA.

[93] Laetz C.A., Baldwin D.H., Hebert V.R., Stark J.D., Scholz N.L. (2014). Elevated temperatures increase the toxicity of pesticide mixtures to juvenile coho salmon. Aquat. Toxicol..

[94] Rehana S., Rajesh M. (2023). Assessment of impacts of climate change on Indian riverine thermal regimes using hybrid deep learning methods. Water Resour. Res..

[95] Staudinger M.D., Lynch A.J., Gaichas S.K., Fox M.G., Gibson-Reinemer D., Langan J.A., Teffer A.K., Thackeray S.J., Winfield I.J. (2021). How does climate change affect emergent properties of aquatic ecosystems?. Fisheries.

[96] Jenkins J.A., Bart H.L., Bowker J.D., Bowser B.P.R., MacMillan J.R., Nickum J.G., Rachlin J.W., Rose J.D., Sorensen P.W., Warkentine B.E. (2014). Guidelines for Use of Fishes in Research—Revised and expanded, 2014. Fisheries.

